# Analysis and prediction of vegetation dynamics under the background of climate change in Xinjiang, China

**DOI:** 10.7717/peerj.8282

**Published:** 2020-01-23

**Authors:** Qingwei Zhuang, Shixin Wu, Xiaoyu Feng, Yaxuan Niu

**Affiliations:** 1 State Key Laboratory of Desert and Oasis Ecology, Xinjiang Institute of Ecology and Geography, Chinese Academy of Sciences, Urumqi, China; 2 College of Earth and Planetary Sciences, University of Chinese Academy of Sciences, Beijing, China; 3 Research Center for Eco-Environmental Sciences, Chinese Academy of Sciences, Beijing, China; 4 College of Resources and Environment, University of Chinese Academy of Sciences, Beijing, China

**Keywords:** Vegetation dynamics, Climate change, Temperature, precipitation, Hydrology, Partial correlation, Greyscale model, prediction, Xinjiang

## Abstract

**Background:**

Vegetation dynamics is defined as a significant indictor in regulating terrestrial carbon balance and climate change, and this issue is important for the evaluation of climate change. Though much work has been done concerning the correlations among vegetation dynamics, precipitation and temperature, the related questions about relationships between vegetation dynamics and other climatic factors (e.g., specific humidity, net radiation, soil moisture) have not been thoroughly considered. Understanding these questions is of primary importance in developing policies to address climate change.

**Methods:**

In this study, the least squares regression analysis method was used to simulate the trend of vegetation dynamics based on the normalized difference vegetation index (NDVI) from 1981 to 2018. A partial correlation analysis method was used to explore the relationship between vegetation dynamics and climate change; and further,the revised greyscale model was applied to predict the future growth trend of natural vegetation.

**Results:**

The Mann-Kendall test results showed that th e air temperature rose sharply in 1997 and had been in a state of high fluctuations since then. Strong changes in hydrothermal conditions had major impact on vegetation dynamics in the area. Specifically, the NDVI value of natural vegetation showed an increasing trend from 1981 to 2018, and the same changes occurred in the precipitation. From 1981 to 1997, the values of natural vegetation increased at a rate of 0.0016 per year. From 1999 to 2009, the NDVI value decreased by an average rate of 0.0025 per year. From 2010 to 2018, the values began an increasing trend and reached a peak in 2017, with an average annual rate of 0.0033. The high vegetation dynamics areas were mainly concentrated in the north and south slopes of the Tianshan Mountains, the Ili River Valley and the Altay area. The greyscale prediction results showed that the annual average NDVI values of natural vegetation may present a fluctuating increasing trend. The NDVI value in 2030 is 0.0196 higher than that in 2018, with an increase of 6.18%.

**Conclusions:**

Our results indicate that: (i) the variations of climatic factors have caused a huge change in the hydrothermal conditions in Xinjiang; (ii) the vegetation dynamics in Xinjiang showed obvious volatility, and then in the end stage of the study were higher than the initial stage the vegetation dynamics in Xinjiang showed a staged increasing trend; (iii) the vegetation dynamics were affected by many factors,of which precipitation was the main reason; (iv) in the next decade, the vegetation dynamics in Xinjiang will show an increasing trend.

## Introduction

As an important part of terrestrial ecosystems, vegetation links among atmosphere, soil and water interactions. It plays an important role in the global biogeochemical and energy cycles (Hao et al., 2016). In terms of environmental protection, vegetation can prevent wind and sand erosion, slow soil erosion, and effectively prevent desertification (Salomao, Silva & Machado, 2019; Zhang et al., 2019a; Zhang et al., 2019b). In terms of the energy cycle, vegetation can effectively reduce the concentration of greenhouse gases, facilitate global carbon balance, and maintain the global climate stability (Ndehedehe & Ferreira, 2019; Servera Vives et al., 2014). It is important to monitor the long-term growth and change of vegetation and to explore its relationship with climate change in the study of global change (Mancino et al., 2013; Shao & Zhang, 2016). In particular, vegetation dynamics are very sensitive to climate change in arid and semi-arid areas (Zhang et al., 2019a; Zhang et al., 2019b; Shao et al., 2019a). Using remote sensing data to dynamically monitor the inter-annual variation of long-term sequence vegetation has become a prevailing research topic (Ernst-Brock et al., 2019; Teh et al., 2015). Some scholars have used different vegetation indices and models to simulate and monitor changes in vegetation dynamics (Cui et al., 2018; Elhakeem & Elshorbagy, 2015; Herrmann et al., 2005). The NDVI has been widely used for research on vegetation dynamics, vegetation productivity measurements, responses to climate change, and desertification (Li & Guo, 2012; Los, 2013; Luan et al., 2013; Wingate, Phinn & Kuhn, 2019). The application of the NDVI value to monitor vegetation dynamics was the basis for better understanding the feedback between vegetation and the atmosphere. Therefore, we used the NDVI of natural vegetation to indicate the response of vegetation dynamics to climate change at regional level.

Global warming has become an indisputable fact (Chapman, 2015; Vale et al., 2018). However, there is no complete assessment of the regional influence under the worldwide climate policy targets. Xinjiang is located in the center of the Eurasian continent. There is an extremely fragile mountain-desert-oasis landscape. The climate has undergone an obvious change, with the average temperature rising significantly and precipitation increasing slightly under the combined influence of global warming and human activities, which may have a huge substantial impact on the natural ecosystems of the region (Chen et al., 2015; Shao, Zhang & Wang, 2017). For example, the plants transpiration and soil moisture consumption may increase in the plain desert areas. This phenomenon may lead to the death of shallow roots (Han et al., 2018). So it is important to monitor vegetation dynamics underground climate change.

In the context of climate change, the monitoring of inter-annual variation vegetation dynamics has become one of the hotspots of ecological, environmental and energy cycle research. In recent research, the NDVI has been widely applied to study the vegetation dynamics and the relationship among vegetation dynamics and climatic factors (Du et al., 2015; Fensholt et al., 2012; Xu et al., 2015; Yan, Wu & Wang, 2013). Most of these scholars directly discussed the correlation between vegetation index and precipitation and temperature. However, vegetation dynamics has been changed under the combined effects of many climatic factors. The cultivated plants, shrubs, and deserts are significantly and positively related to both the increasing maximum temperature and minimum temperature, meanwhile different vegetation types have different responses to asymmetric warming (Ma et al., 2019a; Ma, Xia & Meng, 2019b). The influence of elevation gradients on the start of the growing season, end of the growing season, and the length of growing season on a spatiotemporal scale has been evaluated (Xia et al., 2019). Therefore, in addition to temperature and precipitation, some other climatic factors such as soil moisture, net radiation, and surface specific humidity will be discussed to form a more complete eco-hydrothermal system in this study. In addition, the current research works on vegetation dynamics were analyzed based on historical data. Accurate prediction of the future vegetation dynamics based on the past regular pattern of vegetation dynamics is pivotal for the environmental protection organizations to formulate a reasonable climate policy for climate change.

Xinjiang is particularly vulnerable to climate change (Zhou et al., 2019), and it has experienced significant climate warming in the past 40 years. As a part of the arid region, the vegetation dynamics in Xinxiang are an important ecological barrier to maintain ecological balance (Fang et al., 2018). Over the past few decades, although vegetation restoration appeared in some regions due to numerous vegetation-related programs implemented, vegetation degradation also occurred in several regions due to rapid urbanization. In view of the whole province, the impacts of climatic factors extreme on vegetation dynamics remain unclear in Xinjiang. Therefore, it is crucial to determine the response of vegetation dynamics to climate change by using partial correlation analysis method and Mann-Kendall (MK) test (Sáenz-Romero et al., 2012; Shao et al., 2019b). We will make a reasonable prediction of future vegetation dynamics by constructing a greyscale prediction model in Xinjiang (Zhao et al., 2019). Consequently, our objectives are for following purposes: (1) to discuss the variations of climatic factors; (2) to analyze the spatiotemporal variations of NDVI during 1981-2018 in Xinjiang; (3) to study the relations among climatic factors and NDVI in Xinjiang; (4) to predict and analyze the future vegetation trend from 2019 to 2030. Our study can reveal the precise characteristics of vegetation dynamics in Xinjiang and identify the driving factors that lead to dynamic changes in vegetation. At the same time, this study will provide a theoretical basis for the environmental protection organizations to cope with future climate change and formulate ecological protection policies in Xinjiang.

## Materials & Methods

### Study area

**Figure 1:**
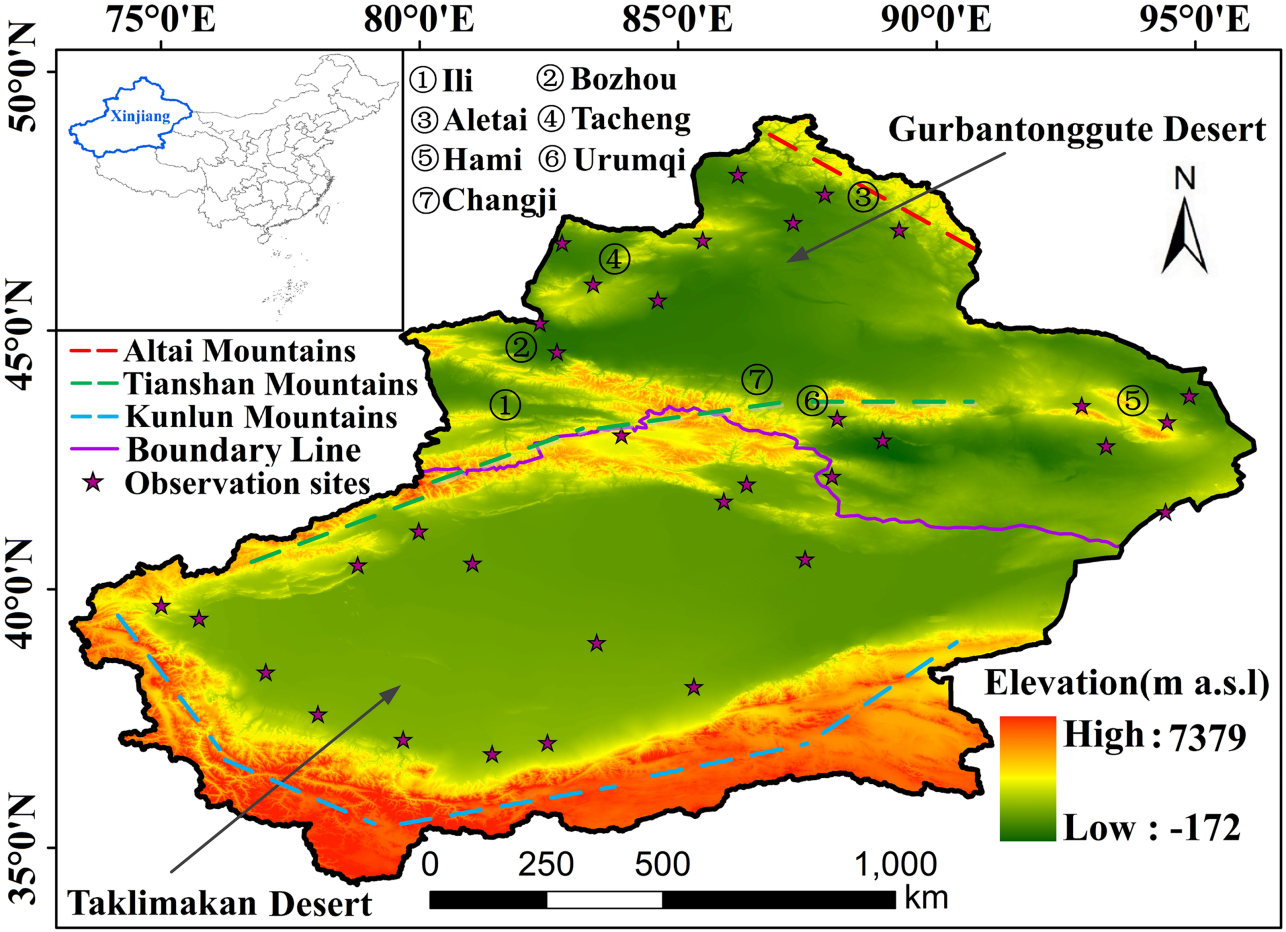
Study area (boundary information was obtained from State Key Laboratory of Desert and Oasis Ecology, Xinjiang Institute of Ecology and Geography, Chinese Academy of Sciences, Approval number: Xinjiang S(2018)033). Three color lines represent three mountains. Purple line represent the boundary of south and north of Xinjiang. Purple stars represent meteorological observation sites. The numbers represent the names of the places which will be mentioned in the text. Map credit: © State Key Laboratory of Desert and Oasis Ecology, Xinjiang Institute of Ecology and Geography, Chinese Academy of Sciences; CC BY NC 4.0.

Xinjiang with an area of approximately 1.66 × 10^4^ km^2^ (75°E–95°E and 35°N–50°N), is located in the center of the Eurasian continent (Fig. 1). It has a geographic location far from the ocean, a complex terrain surrounded by mountains and the geomorphological features of a closed inland basin. These special features have a great influence on the distribution and change of climatic factors in Xinjiang (Du et al., 2015; Wu et al., 2010). The three major mountain ranges in the territory are the Altai Mountains, the Tianshan Mountains and the Kunlun Mountains. The Taklimakan Desert and Gurbantunggut Desert are divided to form a unique landscape pattern of mountains and basins. The climate of Xinjiang is typical of inner-continental land masses with a wide temperature range, low precipitation and low humidity (Wang & Zhang, 2019). The annual precipitation of North Xinjiang is 100 to 500 mm, while that of South Xinjiang is 20 to 100 mm. The annual average temperature in North Xinjiang ranges from 4 °C to 8 °C, and it ranges from 10 °C to 13 °C in South Xinjiang (Luo et al., 2019). There are large areas of forest and grassland vegetation on the Tianshan Mountains, Altai Mountains and Kunlun Mountains. Oases and cities are distributed in the valley plains. In 2018, forest land and grassland accounted for 31.29% of the land area, of which grassland was the main type of vegetation.

### NDVI data

The NDVI dataset from 1981 to 2006 was obtained from the Global Inventor Modeling and Mapping Studies (GIMMS) of the National Aeronautics and Space Administration (NASA) at a spatial resolution of 8 ×8 km and a 15-day temporal resolution. The MoDerate-resolution Imaging Spectroradiometer (MODIS) NDVI dataset from 2000 to 2018 was collected from the United States Geological Survey (USGS) at a spatial resolution of 1 ×1 km and a 16-day temporal resolution. Finally, an NDVI annual dataset with a spatial resolution of 8 ×8 km was obtained. After consistency testing, the R^2^ between the two datasets was 0.96, which was checked by a confidence at the 0.01 level.

### Climatic data

The temperature and precipitation dataset of 33 observation sites from 1981 to 2018 in Xinjiang was obtained from the National Meteorological Information Center of China. We obtained a climate raster dataset whose pixel size and projection was same as GIMMS data by using AUNSPIN to interpolate the climate data. ANUSPLIN is a software Package developed by Australians to simulate surfaces with functions. It has been used to analyze and interpolate multivariate data using a smooth spline function. It can perform reasonable statistical analysis and data diagnosis on data, and can analyze the spatial distribution of data to realize spatial interpolation (Hutchinson, 1998; Xia et al., 2019).

The soil moisture data at different depths, net longwave radiation, net shortwave radiation, and surface specific humidity were collected from the Global Land Data Assimilation System (GLDAS). The GLDAS dataset was estimated against available data from multiple sources. In this study, we used GLDAS 2.0 data (NOAH, 0.25° ×0.25°) for the period 1981–2010 and GLDAS 2.1 data (NOAH, 0.25° ×0.25°) from 2001 to 2018. We built a linear relationship model to extend the GLDAS-2.0 dataset to 2018 by using data from two datasets overlap periods. The depths of the four soil layers ranged from 0 to 10, 10 to 40, 40 to 100, and 100 to 200 cm.

### Land use/Land cover data

The land use/land cover (LULC) data of Xinjiang came from the National Land Use Dataset (NLUD), which was obtained from the Remote Sensing Survey of the Resources and Environment of the Chinese Academy of Sciences. Based on Land satellite (Landsat) MSS/TM/OLI data from America and the China-brazil earth resource satellite (CBERS) from China (Table 1), six major landscape types—cultivated land, woodland, grassland, waterbodies, construction land, and unused land—were extracted from the satellite images through supervised classification (Pan et al., 2019). The classification accuracy was evaluated by overall accuracy and kappa coefficient. The overall accuracy, which represents the probability that the classification result of each random sample is consistent with the actual type of the corresponding region, was the number of all cells that were correctly classified for LULC categories divided by the total number of pixels (Tottrup & Rasmussen, 2004). The kappa coefficient was used to characterize the degree to which the two maps agreed (category maps are consistent with Google Earth high-resolution images or field-measured data or credible classification maps), and it considered both the correct and the incorrect point. When the kappa coefficient was greater than 0.75, we believed that there was a good agreement between the two images (Yang & Xue, 2016).

**Table 1:** Accuracy evaluation of LULC data.

**Year**	**Image source**	**Spatial resolution/m**	**Overall accuracy**	**Kappa coefficient**
1980	Landsat MSS	79	87.50	0.84
1990	Landsat TM	30	90.00	0.86
2000	Landsat TM, CBERS	30, 19.5	92.00	0.89
2010	Landsat TM, CBERS	30, 19.5	90.67	0.88
2018	Landsat OLI	30	92.26	0.90

### Theil-Sen Median analysis and Mann-Kendall test

We have carried out the Mann-Kendall (MK) trend significance test to study the vegetation characteristics through NDVI and climatic factors in Xinjiang (Tong et al., 2018). The MK test, proposed by Kendall and Mann, is a non-parametric test that does not require the data to be normally distributed and has low sensitivity to outliers in the time series (Xiong, Kim & Qiu, 2019). Therefore, this method has been commonly applied to study long-term analysis of meteorological factors and vegetation. This method mainly contains two parameters: Z and *β*. The standard normal test statistic Z, used for testing trend and significance of the time series, is calculated as follows:

\begin{eqnarray*}& & Z= \left\{ \begin{array}{@{}l@{}} \displaystyle \frac{s-1}{\sqrt{Var(s)}} ,S\gt 0 \\ \displaystyle 0,S=0 \\ \displaystyle \frac{s+1}{\sqrt{Var(s)}} ,S\lt 0  \end{array} \right. \end{eqnarray*}

\begin{eqnarray*}& & S=\sum _{i=2}^{n}\sum _{j=1}^{i-1}sign({x}_{i}-{x}_{j}) \end{eqnarray*}

\begin{eqnarray*}& & sign({x}_{i}-{x}_{j})= \left\{ \begin{array}{@{}l@{}} \displaystyle -1,for({x}_{i}-{x}_{j})\lt 0 \\ \displaystyle 0,for({x}_{i}-{x}_{j})=0 \\ \displaystyle 1,for({x}_{i}-{x}_{j})\gt 0  \end{array} \right. \end{eqnarray*}

\begin{eqnarray*}& & Var= \frac{n(n-1)(2n+5)-\sum _{k-1}^{1}{t}_{k}({t}_{k}-1)(2{t}_{k}+5)}{18} \end{eqnarray*}

where *n* is the number of data points, x_i_ and x_j_ are the sequential data in the series, l is the number of tied groups, and t_k_ is the number of data points in the kth group.

In a two-tailed test, the null hypothesis *H*_0_ of no trend should be rejected at the *α* significance level for $ \left\vert Z \right\vert \gt {Z}_{1-\alpha /2}$, which indicates that the time series have a significant variability. The slope (*β*) of Theil-Sen Median indicates that the increasing or decreasing rate of the time series can be obtained using following equation:

$\beta =Median \frac{{x}_{i}-{x}_{j}}{i-j} $ for all *j <i*.

### Partial correlation analysis

Partial correlation only analyzes the degree of correlation between two variables by removing the influence of the remaining variables when one variable is related to multiple variables at the same time. The absolute values of each partial correlation coefficient were categorized as greater than or equal to zero and less than or equal to one. When the absolute value of a partial correlation is equal to one, the two variables are completely related. In contrast, when a partial correlation is equal to zero, the two variables are completely unrelated (Xia et al., 2018).

The statistical significance of the partial correlation coefficient between annual mean NDVI and temperature after other climatic factors was evaluated by the *t*-test as follows:

\begin{eqnarray*}t=r \left[ (n-p-1)/(1+{r}^{2}) \right] ^{1/2} \end{eqnarray*}

where *n* is the total number of years; q is the number of independent variables; r is the partial correlation coefficient between annual mean NDVI and temperature after other climatic factors.

### Greyscale prediction model

Grey system theory (GST), founded by Deng Julong, includes grey system analysis, modelling, prediction, decision-making, control and so on. It has become a useful tool in processing uncertain systems with small samples and poor information (Tian, Minggang & Chuankun, 2014). Grey prediction is an important branch of GST. It can predict the evolution of a system’s behavioral eigenvalues. It can also predict systems that contain both known and uncertain information, such as predicting the ash processes associated with time series that vary within a certain range. It has been widely used in the prediction of risk, minerals, population, water resources, and meteorological factors and so on by grey models (GMs) (Hamzacebi, Karakurt & Effects, 2015; Yang & Xue, 2016; Yuan et al., 2019). Compared with other forecasting methods, only a limited amount of data are needed to estimate the behavior of unknown systems without knowing the mathematical model in the grey system (He et al., 2016).

The equations of the greyscale model are as follows:

(1) The original data are listed as follows:

\begin{eqnarray*}{\mathrm{x}}^{(0)}= \left\{ {\mathrm{x}}^{ \left( 0 \right) } \left( 1 \right) ,{\mathrm{x}}^{ \left( 0 \right) } \left( 2 \right) ,\ldots , \right. \left. {\mathrm{x}}^{ \left( 0 \right) }(\mathrm{n}) \right\} (m=1,2,\ldots ) \end{eqnarray*}

After the accumulation, a new data list is obtained as follows:

\begin{eqnarray*}{\mathrm{x}}^{(\mathrm{m})}= \left\{ {\mathrm{x}}^{ \left( \mathrm{m} \right) } \left( 1 \right) ,{\mathrm{x}}^{ \left( \mathrm{m} \right) } \left( 2 \right) ,\ldots , \left. {\mathrm{x}}^{ \left( \mathrm{m} \right) }(\mathrm{n}) \right\} \right. (m=1,2,\ldots ) \end{eqnarray*}

Find the average and obtain the mean sequence as follows:

*z*^(*m*)^ = {*z*^(*m*)^(2), *z*^(*m*)^(3), …, *z*^(*m*)^(*m*)(*n*)}(*m* = 1, 2, …)

Then the white differential equation corresponding to the grey differential equation of the GM (1, 1) model as follows:

\begin{eqnarray*} \frac{d{x}^{ \left( 1 \right) }}{dt} +\alpha {x}^{ \left( 1 \right) }=\beta \end{eqnarray*}

where *α* is the development coefficient; and *β* is the ash effect.

(2) By solving the white differential equation, the prediction model as follows:

${\mathrm{X}}^{ \left( 1 \right) } \left( \mathrm{k}+1 \right) = \left[ {\mathrm{x}}^{ \left( 0 \right) } \left( 1 \right) - \frac{\beta }{\alpha } \right] {\mathrm{e}}^{-\alpha \mathrm{k}}+ \frac{\beta }{\alpha } $*(k* =1, 2, …*)*

where *α* and *β* are unknown parameters to be determined, and k is a time serial number.

(3) To verify the credibility of the prediction results that were obtained according to the greyscale model, a correlation test was required. In the greyscale model, the correlation coefficient between the measured value and the predicted value at k time was defined as follows:

\begin{eqnarray*}{\xi }_{ij}(k)= \frac{{\Delta }_{min}+\rho {\Delta }_{max}}{{\Delta }_{ij}(t)+\rho \Delta \text{_}max} \end{eqnarray*}

The overall correlation between the measured value and the predicted value was:

\begin{eqnarray*}{R}_{ij}(k)& = \frac{1}{n-i} \sum _{k=1}^{n-1}{\xi }_{ij}(k) \end{eqnarray*}

\begin{eqnarray*}{\Delta }_{ij} \left( k \right) & = \left\vert {x}_{i} \left( k \right) -{x}_{j}(k) \right\vert \end{eqnarray*}

where ${\Delta }_{ij} \left( \mathrm{k} \right) $ is the absolute difference between the measured value and the predicted value at k time; Δ_*min*_ is the minimum value of the absolute difference; Δ_*max*_ is the maximum value of the absolute difference; *ρ* = 0.5 is the resolution coefficient.

## Results

### Spatiotemporal changes in the climatic factors

**Figure 2:**
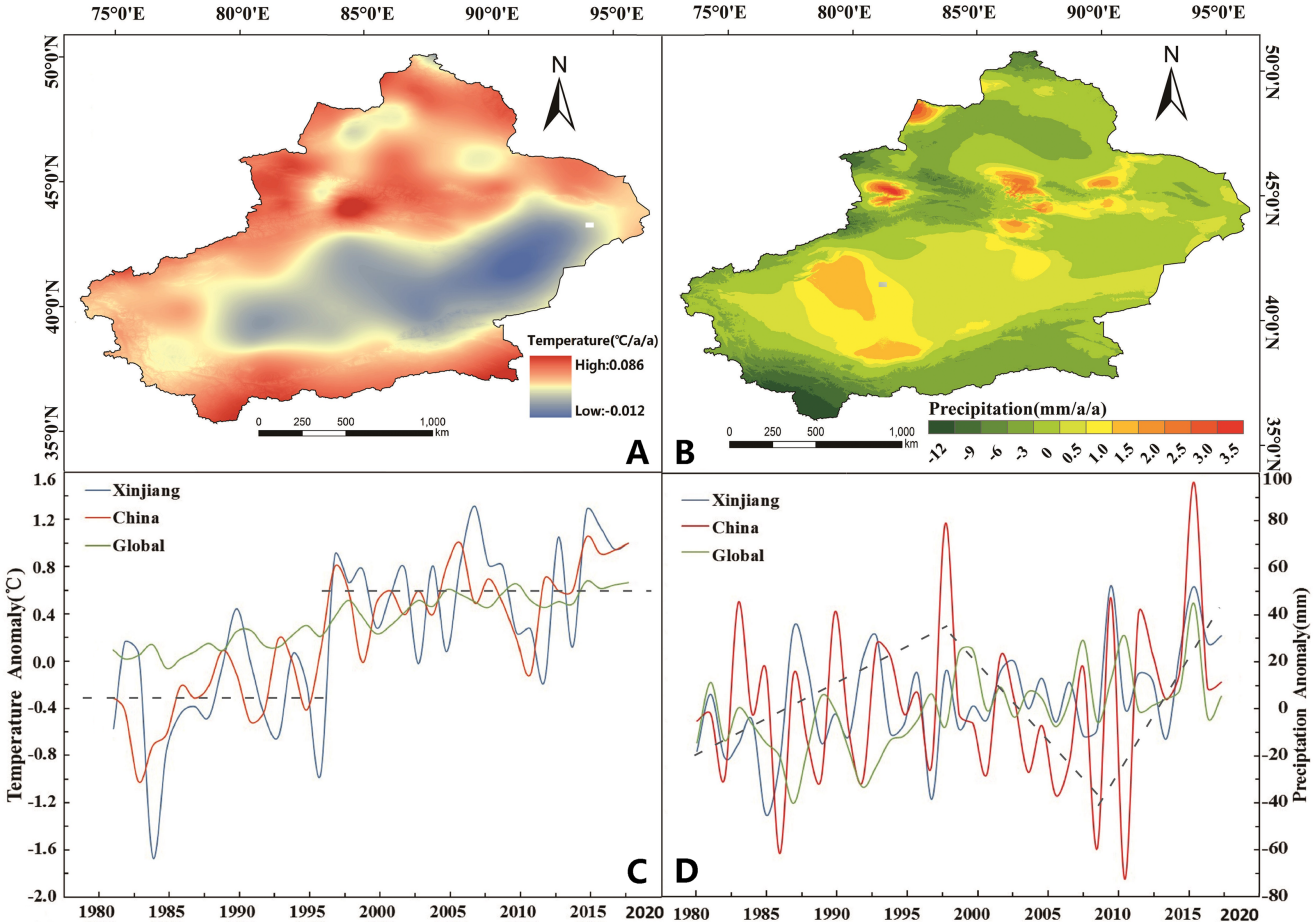
The spatiotemporal changes in temperature and precipitation during 1981–2018. (A) Trend of temperature in Xinjiang. (B) Trend of precipitation in Xinjiang. (C) Annual temperature anomalies of Globe, China and Xinjiang. (D) Annual precipitation anomalies of Globe, China and Xinjiang. Map credit: © State Key Laboratory of Desert and Oasis Ecology, Xinjiang Institute of Ecology and Geography, Chinese Academy of Sciences; CC BY NC 4.0.

The spatial distribution of temperature trends indicated that the areas with accelerated warming were mainly concentrated in the southern margin of the Altai Mountains, the north and south slopes of the Tianshan Mountains and the northern margin of the Kunlun Mountains (Fig. 2A). From 1981 to 2018, the annual temperatures in Xinjiang showed a clear upward trend at a rate of 0.328/10 years (0.01 confidence level) (Table 2). In 1997, there was a sharp increase in the temperature change trend in Xinjiang according to the results of MK test, and it has remained at a high level since then (Table 2, Fig. 2C).

**Table 2:** Trend test of NDVI and climatic factors in Xinjiang in different periods.

**Indicator**	**1981–2018**	**1981–1997**	**1998–2018**
Annual NDVI (/10a)	0.008^**^	0.006^**^	0.000
0–10 cm (Kg/(m^2^.10a))	0.351	−0.782^**^	1.010^**^
10–40 cm (Kg/(m^2^.10a))	2.525^**^	−0.503^*^	5.020^**^
40–100 cm (Kg/(m^2^.10a))	6.097^**^	0.412^*^	11.042^**^
100–200 cm (Kg/(m^2^.10a))	10.008^**^	−0.198	18.743^**^
Net Radiation (W/(m2.10a))	2.243^**^	6.900^**^	−0.301^*^
Specific Humidity (10^−3^/10a)	−0.223^**^	−0.254^**^	−0.470^*^
Precipitation (mm/10a)	14.482^**^	−11.517^*^	10.590^*^
Temperature (°C/10a)	0.328	0.539^*^	0.252

**Notes.**

* Significant at the 0.05 level.

** Significant at the 0.01 level.

The spatial distribution of precipitation trends from 1981 to 2018 indicated that areas with significant increases in precipitation concentrated in the Urumqi, Changji, Ili, Bozhou, and southwestern Taklimakan Deserts (Fig. 2B). From 1981 to 2018, the precipitation showed a slightly increasing trend in Xinjiang with a rate of 14.482 mm/10 years (0.01 confidence level) (Table 2). Moreover, the decadal fluctuation amplitude was greater in Xinjiang than the overall global level. The annual precipitation in Xinjiang increased from 1981 to 1997 at a rate of −11.517 mm/10 years (0.05 confidence level) (Table 2). The results of MK test indicated that it reached a peak in 1998. From 1998 to 2018, the annual precipitation trend showed an increasing trend with a rate of 10.590 mm/10 years (0.05 confidence level) (Table 2, Fig. 2D).

**Figure 3:**
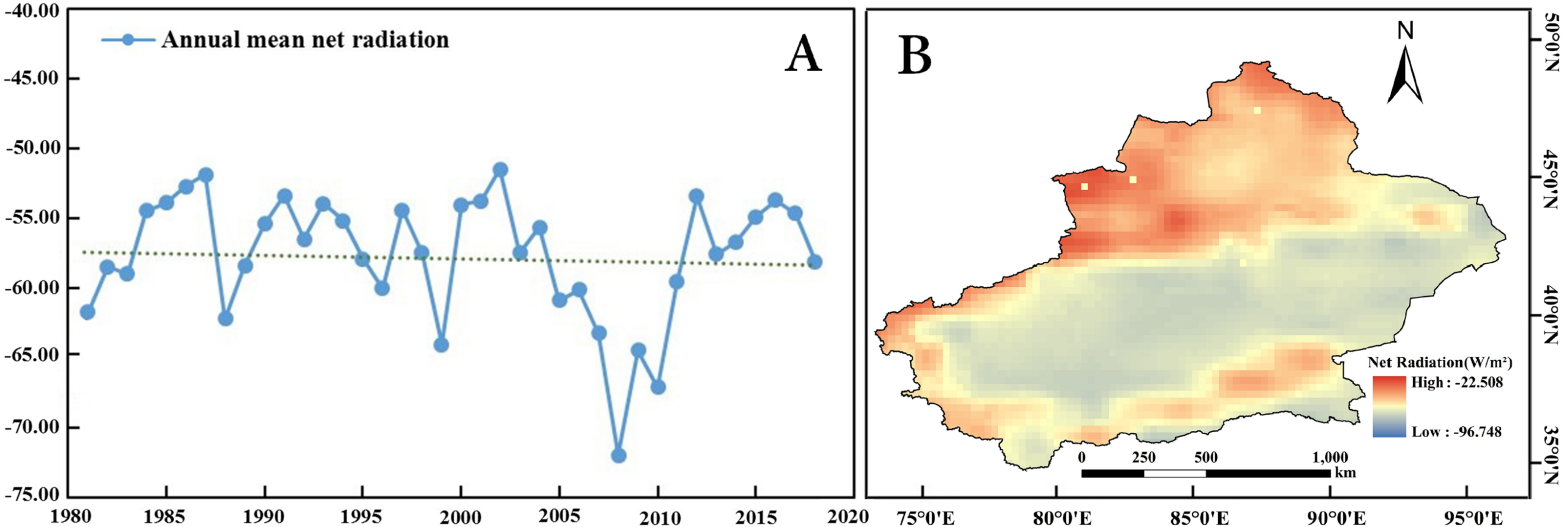
Spatiotemporal changes in the annual net radiation in Xinjiang from 1981 to 2018. (A) Anomalies in the annual net radiation in Xinjiang. (B) Distribution of the net radiation in Xinjiang. Map credit: © State Key Laboratory of Desert and Oasis Ecology, Xinjiang Institute of Ecology and Geography, Chinese Academy of Sciences; CC BY NC 4.0.

The annual mean net radiation significantly (*p* < 0.01) increased at a rate of 2.243W/10 years in Xinjiang during 1981 to 2018 (Table 2, Fig. 3A). From Fig. 3B, it was observed that the net radiation showed large regional differences, increasing from the south to the north of Xinjiang. The high net radiations were detected in the Gurbantonggute Desert, Bozhou and Ili region.

**Figure 4:**
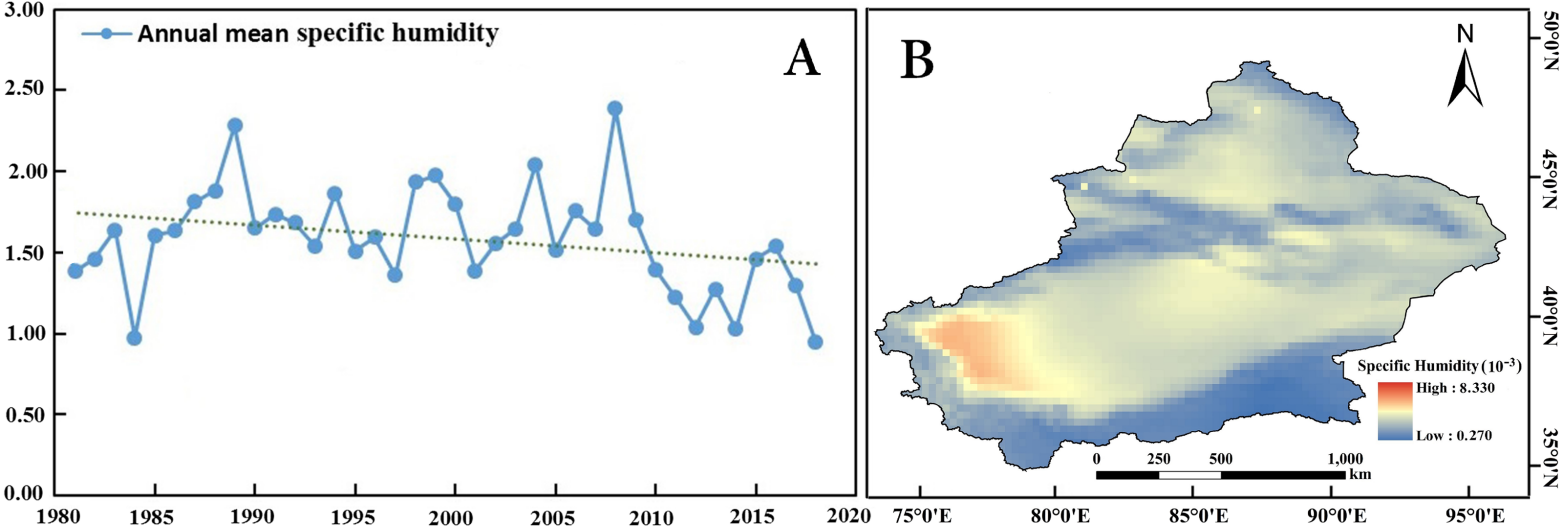
Spatiotemporal changes in the annual specific humidity in Xinjiang from 1981 to 2018. (A) Anomalies in the annual specific humidity in Xinjiang. (B) Distribution of the specific humidity in Xinjiang. Map credit: © State Key Laboratory of Desert and Oasis Ecology, Xinjiang Institute of Ecology and Geography, Chinese Academy of Sciences; CC BY NC 4.0.

The annual mean specific humidity showed a decreasing trend at a rate of 0.223 ×10^−3^/10 years (0.01 confidence level) during 1981 to 2018 (Table 2, Fig. 4A). Figure 4B indicates that there are significant regional differences in the annual precipitation variation trend. The low specific humidity regions were mainly located in the three mountains (including Altai Mountains, Tianshan Mountains and Kunlun Mountains).

**Figure 5:**
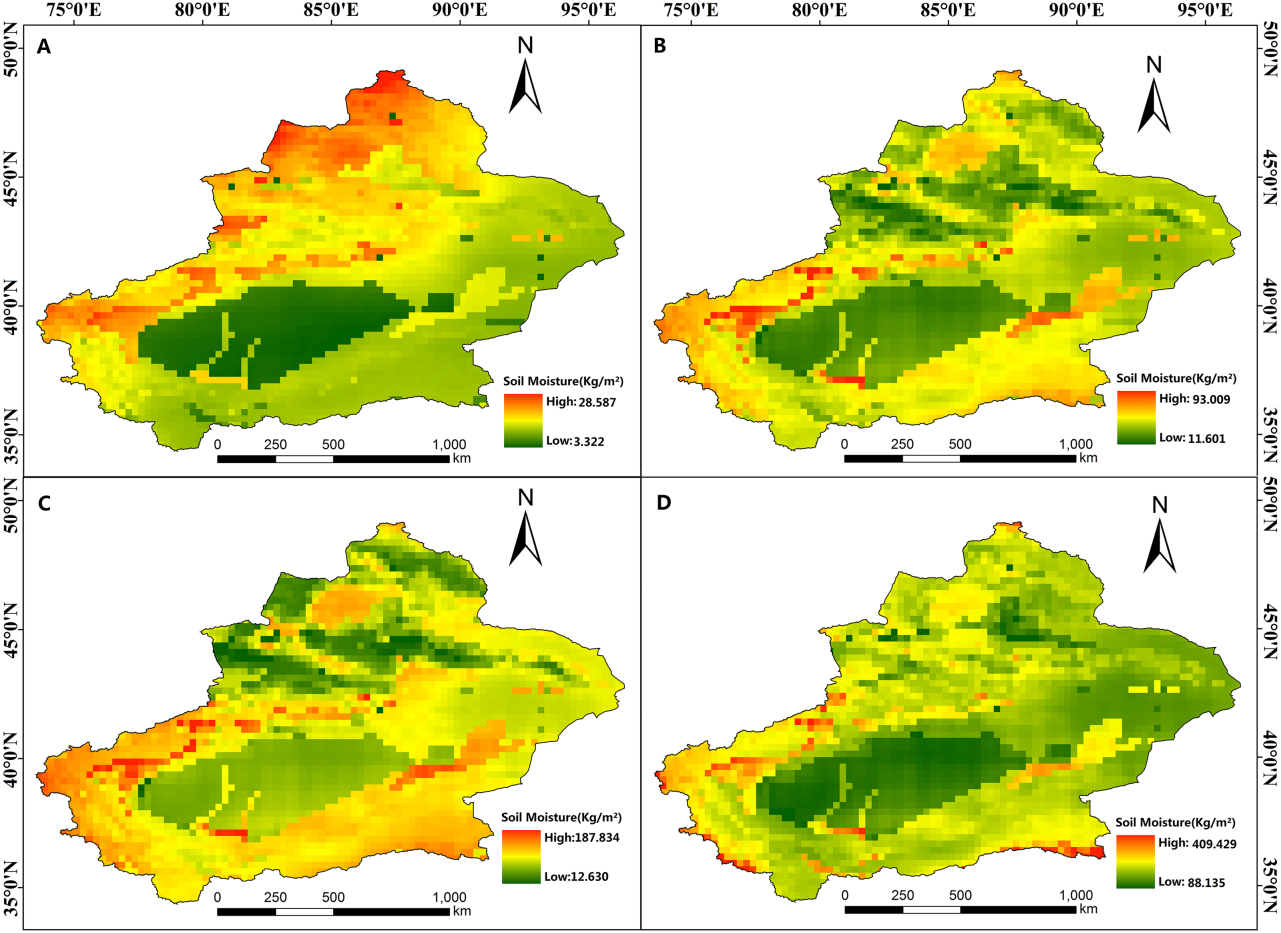
Average soil moisture at different depths from 1981 to 2018. (A) 0–10 cm; (B) 10–40 cm; (C) 40–100 cm; (D) 100–200 cm. Map credit: © State Key Laboratory of Desert and Oasis Ecology, Xinjiang Institute of Ecology and Geography, Chinese Academy of Sciences; CC BY NC 4.0.

The soil moisture at different depths showed different variations in Xinjiang. The soil moisture at 0–10 cm showed an increasing trend at a rate of 0.351 kg/ (m^2^.10 years) during 1981 to 2018, but did not reach a significant level (*p* = 0.072). The soil moisture at other depths significantly (*p* < 0.01) increased at different rates. There were similarities at the four depths, such as the low soil moisture regions were mainly located in two deserts (including Gurbantonggute Desert and Taklimakan Desert) (Fig. 5). In the horizontal distribution, the shallow soil moisture in the southern Tianshan Mountains was significantly lower than that in the north of the Tianshan Mountains (Fig. 5A). In sub-shallow soils, the soil moisture in the Taklimakan Desert and the Gurbantunggut Desert was significantly lower than that in other regions (Figs. 5B and 5C). In the deep soil, the soil moisture was lower overall in Xinjiang, and the Taklimakan region had the lowest soil moisture (Fig. 5D).

### Spatiotemporal changes in the annual mean NDVI

**Figure 6:**
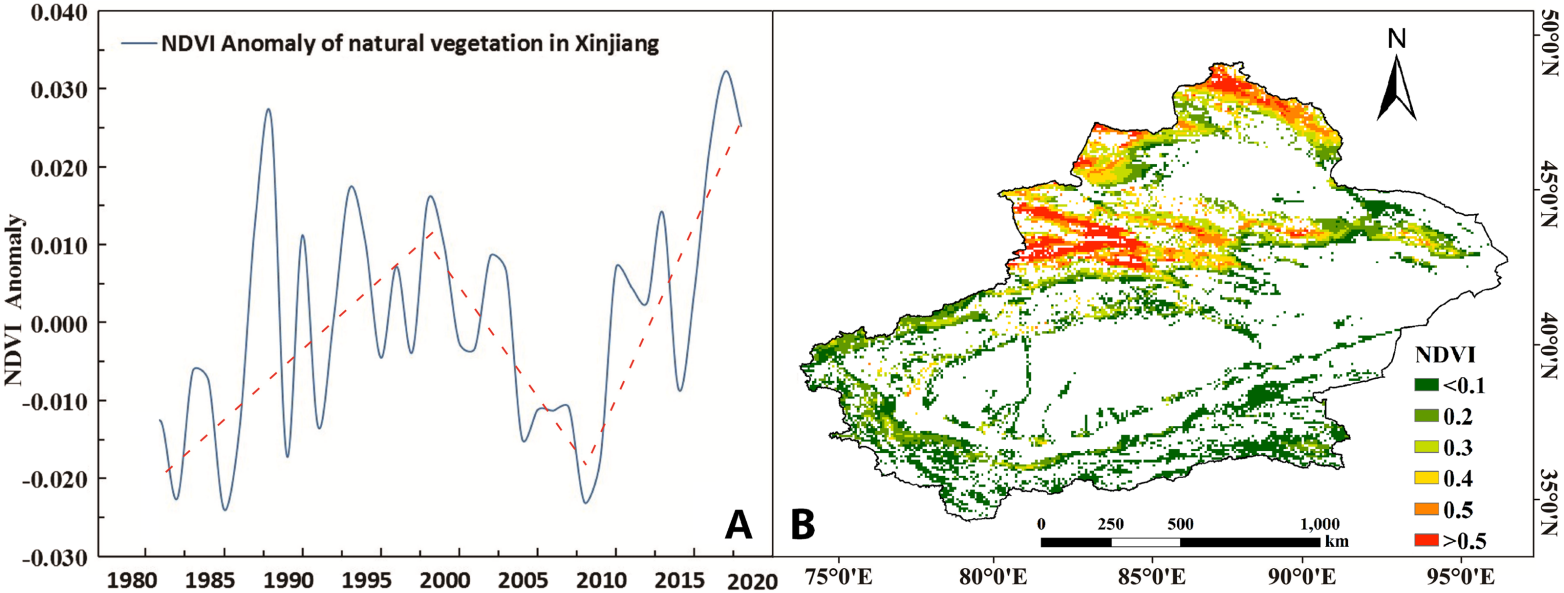
Spatiotemporal changes in the annual NDVI in Xinjiang from 1981 to 2018. (A) Anomalies in the annual NDVI of natural vegetation in Xinjiang. (B) Distribution of the natural NDVI in Xinjiang. Map credit: © State Key Laboratory of Desert and Oasis Ecology, Xinjiang Institute of Ecology and Geography, Chinese Academy of Sciences; CC BY NC 4.0.

We clipped the NDVI raster data to obtain the distribution of natural vegetation NDVI by using the LULC data to create a mask. The NDVI annual average anomaly of natural vegetation can be obtained by statistics (Fig. 6A). During the entire study period, the annual NDVI value of natural vegetation showed an increasing trend with a rate of 0.008/10a (0.01 confidence level) (Table 2). The trend of NDVI was consistent with the annual change trend of precipitation with a characteristic of “firstly increasing, then decreasing and finally increasing”. From 1981 to 1997, the NDVI value of natural vegetation increased at an annual rate of 0.0016. However, the growth trend has reversed since 1998. From 1999 to 2009, the NDVI value decreased at an average rate of 0.0025 per year. From 2010 to 2018, the NDVI value showed an increasing trend with an average annual growth rate of 0.0033 and reached a peak in 2017.

The vegetation in Xinjiang is dominated by desert vegetation mixed with alpine and oasis vegetation patches. The NDVI annual value was calculated by the maximum synthesis method. In the longitudinal distribution, the distribution of the NDVI showed a gradual decrease from the west to the east, influenced by the westerly circulation from 1981 to 2018. On the whole, its distribution characteristic was consistent with the distribution characteristic of sub-shallow soil moisture. In the latitude distribution, the NDVI values of vegetation in northern Xinjiang were generally higher than the NDVI value of vegetation in southern Xinjiang. The high vegetation dynamics areas were mainly concentrated in the north and south slopes of the Tianshan Mountains, the Ili River Valley and the Altay area as these areas had relatively more precipitation (Fig. 6B).

### Correlation between NDVI and climatic factors

To further examine the impacts of climatic factors on vegetation in Xinjiang. We used partial correction coefficients to determine the relationship between annual mean NDVI and climatic factors (Table 3). The annual mean NDVI was significantly and positively correlated with average soil moisture at 10–40 cm, 40–100 cm, 100–200 cm and precipitation during 1981 to 2018. However, there was not significant correlation between annual NDVI and average soil moisture at 0–10 cm, net radiation, specific humidity and temperature. Table 3 showed that the annual mean NDVI was most significant and positively correlated with precipitation (exceeding the 0.01 confidence level), whereas it was negative correlated with specific humidity (not reach significance level, *R*^2^ =  − 0.217).

**Table 3:** Relationships among annual NDVI and average soil moisture, net radiation, specific humidity, precipitation and temperature in Xinjiang from 1981 to 2018.

**Indicator**	**NDVI**		
	** 1981–2018**	**1981–1997**	**1998–2018**
0–10 cm	0.141	−0.054	0.123
10–40 cm	0.494^**^	0.304	0.616^**^
40–100 cm	0.502^**^	0.568^*^	0.631^**^
100–200 cm	0.474^**^	0.067	0.617^**^
Net radiation	0.264	0.070	0.459^*^
Specific humidity	−0.217	0.131	−0.417
Precipitation	0.699^**^	0.727^**^	0.666^**^
Temperature	0.164	−0.039	0.177

**Notes.**

* Significant at the 0.05 level.

** Significant at the 0.01 level.

NDVI natural vegetation annual NDVI

Average Soil Moisture at 0–10 cm; 10–40 cm, Average Soil Moisture at 10–40 cm; 40–100 cm, Average Soil Moisture at 40–100 cm; 100-200 cm, Average Soil Moisture at 100–200 cm.

In different periods, there were obvious variations in the relationships between annual mean NDVI and climatic factors. From 1981 to 1997, the overall relationship between annual mean NDVI and climatic factors was lower than the level of the whole study period (Table 3). Only two factors (soil moisture at 40–100, *R*^2^ = 0.568, exceeding the 0.05 confidence level; precipitation, *R*^2^ = 0.727, exceeding the 0.01 confidence level) reached significant level. The soil moisture at 0–10 cm and temperature were negative correlated with the annual mean NDVI (*R*^2^ = −0.054, −0.039, respectively). From 1998 to 2018, the overall relationship between annual mean NDVI and climatic factors was obviously higher than the level of the whole study period (Table 3). The soil moisture at 10–40 cm, 40–100 cm, 100-200 cm and precipitation exceeded the 0.01 confidence level (*R*^2^ = 0.616, 0.631, 0.617, 0.666, respectively), while the net radiation reached 0.05 confidence level (*R*^2^ = 0.459). However, there was no significant correlation between the annual NDVI and soil moisture at 0–10 cm, specific humidity and temperature during 1998–2018. The results showed that the relationship between annual NDVI and precipitation was highest in any periods, so we can determine that precipitation was the main factor affecting vegetation dynamics.

### Prediction of the natural vegetation dynamics

The values of the parameters *α* and *β* were calculated to be −0.002424 and 0.295315, respectively. Thus, the prediction model was ${\mathrm{X}}^{ \left( 1 \right) } \left( \mathrm{k}+1 \right) =122.131814{\mathrm{e}}^{0.002424\mathrm{k}}-121.845414$*.* According to experience, we let *ρ* = 0.5, and then made a relevance test for the model. If the degree of correlation is greater than 50%, we believed that the credibility passed the test. The results showed that the similarity between the measured value and the predicted value was 98.28% (Table 4).

**Table 4:** Accuracy evaluation between exited data and predictive data.

**Year**	**Exited value**	**Predictive value**	**Difference**	**Similarity (%)**
1981	0.2876	0.2854	−0.0022	99.23%
1990	0.3134	0.3030	−0.0104	96.68%
2000	0.2996	0.3010	0.0014	99.53%
2010	0.3092	0.3183	−0.0091	97.06%
2018	0.3174	0.3209	0.0035	98.90%

**Figure 7:**
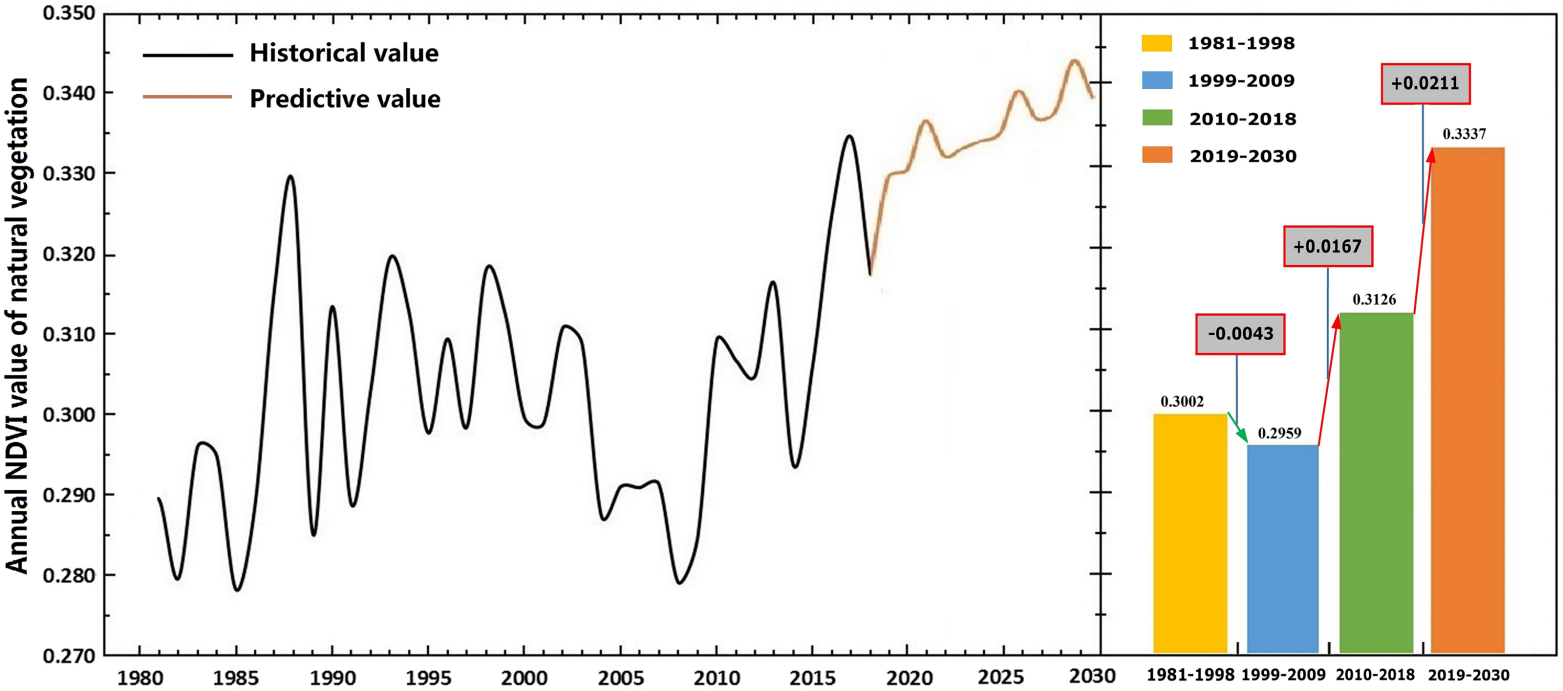
Temporal variation in average annual NDVI of natural vegetation from 1981 to 2030. The black line on the left indicates historical data. The brown line represents predicted value. The histogram shows the average of NDVI over different periods. The numbers in the box represent the ranges of change in the average NDVI.

The predicted results demonstrated that the average annual NDVI of natural vegetation fluctuated from 2019 to 2030 in Xinjiang, and the average value in 2030 increased by 0.0196 relative to that in 2018 with an increase of 6.18%. The annual average NDVI of natural vegetation reaches 0.3337 during the period 2019 to 2030. Figure 7 shows that 1999–2009 was the worst decade for vegetation growth, the annual average of NDVI was only 0.2959 during this period. This phenomenon occurred under the combined effect of climatic factors. Statistics showed that soil moisture at different depths and precipitation during this period were lower than the entire study period, temperature was higher than the entire level (Table 2). After 2010, vegetation dynamics showed a steadily improving trend with an average annual rate 0.0026 under the influence of climate and environmental conditions.

## Discussion

### Trends of vegetation dynamics

In section “Spatiotemporal Changes in Annual Mean NDVI”, we found that the annual mean NDVI showed an increasing trend in Xinjiang during 1981 to 2018. This result was similar to previous studies. Ma et al. (2019a) suggested that vegetation variability to asymmetric warming is important to understand the changes in vegetation photosynthetic activity under global warming and ultimately influences regional and hemispheric-scale carbon balances. Cui et al. (2019) found that the annual mean NDVI over the whole Yangtze River Basin showed a significantly increasing trend during 1982–2015. Spatially, annual mean NDVI significantly increased in the northern, eastern, and parts of southwestern Yangtze River Basin, while it decreased in the parts of southern YRB. Yao et al. (2019) showed that greening speed may be slower in arid than in humid regions, and in urban cores than in rural areas. Thus some benefits from vegetation greening may be much less in arid than in humid regions, and in urban cores than in rural areas.

Du et al. (2016a) and Du et al. (2016b) showed that the vegetation greenness in growing season (including spring, summer, and autumn) increased significantly from 1982 to 2012 in Xinjiang. The NDVI in growing season increased significantly from 1982 to 1998, then decreased significantly from 1998 to 2012; this trend was also observed in summer and autumn seasons. Similar to the changes in greenness at regional scales, the percentages of land areas experiencing positive anomalies also increased significantly during 1982 to 2012. Xu et al. (2015) found that the NDVI values in growing season were high in the west and northwest and low in the south and southeast. The area with increased NDVI values was much larger than the area with decreased NDVI values during the study period in Xinjiang. The values of NDVI in the cropland area significantly increased whereas the values of NDVI in the grassland area significantly decreased. The center of vegetation moved from north to south in the whole study area, moved from southwest-northeast in the north to the Tianshan Mountains and moved from west to east in the south to the Tianshan Mountains.

### The effects of climate change on NDVI

Previous studies showed that climate is the major factor influencing the vegetation dynamics (Chen et al., 2015; Li et al., 2015). Qu et al. (2018) showed that temperature is a controlling factor determining the vegetation greenness in the Yangtze River Basin, and the response of vegetation to precipitation is relatively lower because of the abundant water. Xia et al. (2019) suggested that the variation in vegetation responses to diurnal temperature changes is important for understanding the changes in vegetation photosynthetic activity in a warming world. Meanwhile, land use changes caused by ecological restoration project is the major driving factor for improving vegetation conditions in YRB, and the spatial distributions between human-induced GSN increasing trends and areas with increased forest have a strong consistency in the north of YRB. In this study, we discussed the correlation between NDVI and climatic factors.

The results showed that there was significant correlation between precipitation and annual NDVI during different periods. Herrmann et al. (2005) showed that vegetation dynamics in the African Sahel was most significant correlated with precipitation and human activities. Richard & Poccard (1998) showed that precipitation was highly correlated with NDVI in Southern Africa.

There was not significant correlation between annual NDVI and temperature in this study (Table 3). Dai, Zhang & Wang (2010) showed that there was a significant linear relationship between vegetation cover and the monthly average temperature and precipitation during the years. However, Peng et al. (2011) showed that growing season NDVI was significantly correlated with temperature in the central and east China. A widely accepted viewpoint is that vegetation growth in southern China is mainly affected by temperature, while precipitation is not a limitation for vegetation growth.

There were few studies that focused on the relationship between net radiation (specific humidity) and annual NDVI. Our results indicated that they were not significantly correlated with annual NDVI, but they were important factors influencing the vegetation dynamics.

Consistent with previous studies, our results showed that the soil moisture at different depths was significantly and positively correlated with annual NDVI except the soil moisture at 0–10 cm, and the significance was different at different depths in different periods. Water is the limiting factor for ecological attributes in arid and semiarid areas, and vegetation dynamics are highly sensitive to alterations in water availability. This indicates that water conveyance is necessary for protecting important vulnerable ecological regions (Alejandro Saiz-Rodriguez et al., 2019). Peterman et al. (2014) showed that annual was very sensitive to soil depth in simulations of carbon and hydrological variables.

### Credibility of the predicted results

In recent studies, some predictive models have been used to predict vegetation dynamics. Bai et al. (2019) used pixel trend extrapolation model to predict the vegetation dynamics. The results suggest that the pixel trend extrapolation model could yield a reasonable result. Zhu et al. (2019) used the statistical prediction model to vegetation index in the Three-Rivers-Source Region. Du et al. (2016a) and Du et al. (2016b) used CA-Markov model to predict the vegetation coverage condition in Shijiazhuang.

There were two main methods for verifying the greyscale prediction results. The first was the posterior difference test. The posterior difference ratio was obtained through the standard deviation of the original data column divided by the standard deviation of the residual data column. The smaller the posterior difference was that the closer the estimated value was to the actual value. The posterior difference test method has been applied by many scholars because of its simple and easy-to -calculate characteristics. The correlation degree of the current prediction result was 98.28%, which was in accordance with the confidence requirement.

The prediction results of this study showed that the vegetation dynamics fluctuated upwards in Xinjiang from 2019 to 2030, and the vegetation growth status has been continuously improved. This result was consistent with the results of many scholars in Xinjiang and even globally (LeVine & Crews, 2019; Zhu et al., 2016). It was also in line with our understanding on the ground.

### Study limitations

Although we have performed a great deal of work, there were still some shortcomings in this study. Regarding the data, the NDVI data were collected from different sources, which have different spatial and temporal resolutions. Although interpolation had been performed, a linear regression model was established for fitting, and the accuracy test was passed, but there may still be a small amount of deviation. In terms of research methods, the annual values of various climatic factors and NDVI values were discussed, but they may not have been elaborated on the time scale. In addition, we used existing vegetation dynamics data the annual average of all existing NDVI values to predict future data. The credibility of the forecast results can only be ensured on an existing basis. The reverse feedback process of future changes to existing results cannot be ensured. Finally, the research on the relationships between vegetation dynamics and soil moisture at different depths were discussed not deep enough. Later, a further study could be carried out based on the type of vegetation, the depth of the vegetation roots, the type of soil and the topography of the study area.

## Conclusions

Our results suggested that the climate has undergone tremendous changes in the past four decades in Xinjiang. The temperature rose sharply in 1997 and then remained at a high level, meanwhile the precipitation in Xinjiang showed a trend of rising volatility. The other climatic factors have also changed to varying degrees. The vegetation dynamics in Xinjiang showed obvious volatility, and those in the end stage of the study were higher than the initial stage the vegetation dynamics in Xinjiang showed a staged increasing trend. The vegetation dynamics were affected by many factors, of which precipitation was the main reason (exceeding the 0.01 confidence level, *R*^2^ = 0.666). The prediction results of vegetation dynamics indicated that the annual average NDVI value of natural vegetation in Xinjiang showed a fluctuating upward trend from 2019 to 2030. This study comprehensively analyzed the effects of climatic factors such as temperature, precipitation, soil moisture, net radiation and specific humidity on vegetation dynamics. It is important for how to deal with regional climate change.

##  Supplemental Information

10.7717/peerj.8282/supp-1 Supplemental Information 1 Raw data

10.7717/peerj.8282/supp-2 Supplemental Information 2 The anuspline results

10.7717/peerj.8282/supp-3 Supplemental Information 3 The accuracy of the extended GLDAS 2.0 dataset

## Additional Information and Declarations

### Data Availability

The following information was supplied regarding data availability: The climatic dataset was obtained from the National Meteorological Information Center of China (http://data.cma.cn), and is available in the Supplemental Files.
