# Supplementary material for: Analysis and prediction of vegetation dynamics under the background of climate change in Xinjiang, China
Source: PeerJ. 2020 Jan 23;8:e8282. doi: 10.7717/peerj.8282 (PMC6983299; doi:10.7717/peerj.8282)
Supplement: Supplemental Information 1 [file peerj-08-8282-s001.zip › Introduction of data/Evaluation Report of China ground precipitation 0.5°×0.5° grid dataset.docx]

**Evaluation Report of China ground precipitation 0.5°×0.5° grid dataset (V2.0)**

**National Meteorological Information Center**

**August 2012**

1. **Overview**

How to transform discrete meteorological station data into regular grid data through appropriate spatial interpolation methods is of great significance for climate change analysis and numerical simulation research. The ground-based meteorological data construction project completed the quality inspection, control and correction of 2474 national-level ground stations' basic meteorological data, and formed a set of high-quality, national-level and provincial-level ground-based basic data files. The Meteorological Data Room of the National Meteorological Information Center uses the thin-strip spline method on the basis of the ground-based data file precipitation data. The digital elevation data is added to eliminate the influence of elevation on the spatial interpolation accuracy of precipitation under the unique terrain conditions in China. The daily ground precipitation value and the monthly value of 0.5°×0.5° grid data set since 1961 are established. The data base is described by describing trends and magnitudes of precipitation changes in China.

This paper mainly analyzes and evaluates the precipitation grid dataset from three aspects: interpolation error analysis, precipitation climatic state spatial characteristics, typical case analysis and interpolation test. The main conclusions are as follows: Firstly, the spatial distribution characteristics and seasonal characteristics of the evaluation indicators such as cross-validation, root mean square error, absolute error and relative error are given. The results show that the lattice analysis value is highly correlated with the original sequence and the error is small. “China's ground precipitation 0.5°×0.5° grid data set (V2.0)” can accurately reflect the changing characteristics of precipitation. Second, the latticed precipitation data after interpolation can describe the main spatial characteristics of the annual average precipitation field in the southeast and the northwest in China in a detailed and accurate way. Thirdly, the analysis of precipitation from the perspective of surface rainfall shows that although grid processing has certain difficulty in accurately depicting precipitation anomaly samples (such as large precipitation), when considering the amount of surface rainfall within a certain range, grid points The difference between the analytical value and the observed value is small, and the latticed precipitation can well describe the magnitude of the surface rainfall within a certain range.

1. **Materials and Methods**
   1. **Materials**

The data sources for the development of “China Ground Precipitation 0.5°×0.5° Grid Dataset (V2.0)” include:

1. The monthly and daily precipitation data of 2474 national-level stations in the country archived by the meteorological data room. The data comes from the monthly report of the surface meteorological records reported by the climate data processing departments of the provinces, municipalities and autonomous regions. The data is collected and sorted by the National Meteorological Information Center's basic data, and undergoes rigorous inspection and review.
2. The Chinese range 0.5°×0.5° digital elevation model data DEM generated by Gsample 030 data (resolution 30m×30m) is resampled.


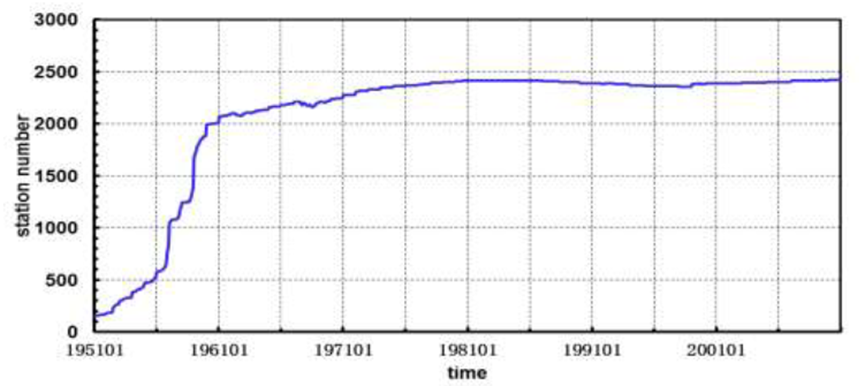


Figure 1 The variation curve of the number of stations on the ground high-density stations in China from 1951 to 2010.


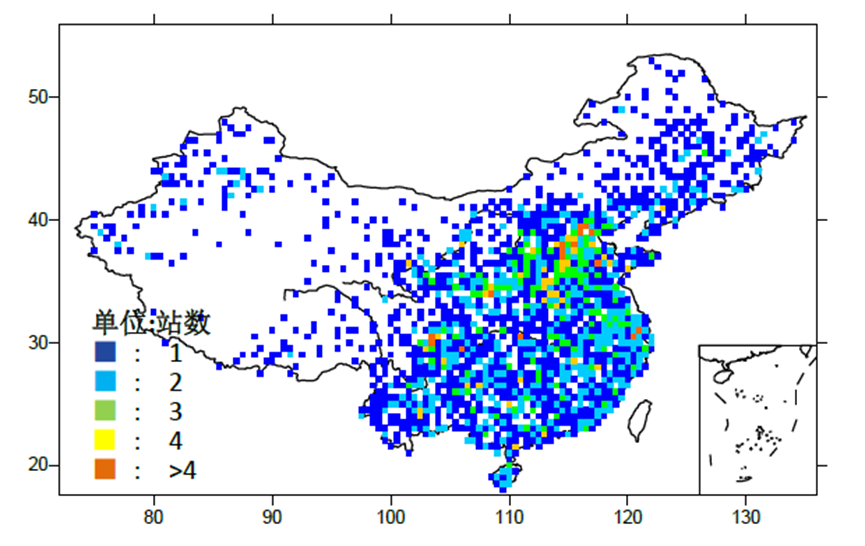


Figure 2 Number of stations in the national unit grid (0.5° × 0.5°) in 2010.

- 1. **Lattice method**

Spatial interpolation can be implemented in general GIS software, but there are not many systems that specifically target climate data and can take into account accuracy, convenience, and time series characteristics. Thin Plate Spline (TPS) only uses spatial distribution as a function of observation data without the need of prior knowledge and physical processes, which effectively improves the accuracy of interpolation.

Partial thin plates smoothing splines are an extension of the thin-strip smooth spline prototype, which allows the introduction of linear covariate sub models, such as temperature and altitude, precipitation and coastline, in addition to the normal spline arguments. Relationships, etc. The theoretical statistical model of the local thin disk smooth spline is expressed as follows:

Figure 3 Data set production flow chart.


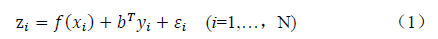


Z_i_ is the dependent variable at the point i of the space; xi is the d-dimensional spline independent variable, f is the unknown smooth function for xi to be estimated; y_i_ is the p-dimensional independent covariate; b is the p-dimensional coefficient of y_i_; ε_i_ is The independent variable random error with the expected value of 0 and the variance is wiσ2; w_i_ is the known local relative coefficient of variation as a weight, and σ2 is the error variance, which is constant at all data points, but is usually unknown.

It can be seen from equation (1) that when the second term is missing in the formula, ie, the covariate (p = 0), the model is reduced to a thin disk smooth spline prototype; when the first independent variable is missing, the model becomes multivariate linear Regression (this is not allowed in ANUSPLIN). In fact, the thin disk spline function can be understood as a generalized standard multivariate linear regression model, except that its parameters are replaced by a suitable nonparametric smoothing function. The function f and the coefficient b are determined by a least squares estimate:


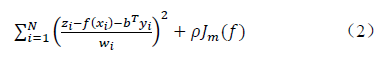


Where Jm(f) is the roughness measure function of the function f(xi), defined as the m-order partial derivative of the function f (called the number of splines in ANUSPLIN, also called the number of roughness times), and ρ is a positive smooth parameter. The balance between data fidelity and surface roughness is usually determined by the minimization of generalized cross validation (GCV), or the maximum likelihood method (GML) or expected real square error. MSE (expected true square error) minimizes the determination. The GCV calculation can use the "one point move" method, which sequentially removes a sample point, and uses the remaining sample points to perform surface fitting under a certain smooth parameter to obtain the estimated value of the point, and then calculate the observed value and the estimated value. Variance. ANUSPLIN also provides a method for judging the selection of smoothing parameters for both GCV and GML.

- 1. **Inspection method and evaluation index**

In order to test the interpolation effect of the interpolation method and the interpolation model on the actual temperature field, the generalized cross-validation method is used for evaluation. The principle is to first assume that the meteorological element value of each site is unknown, estimate it with the value of the surrounding site, and then judge the merits of the interpolation method based on the error of the actual observation value and the estimated value of all stations. The GCV consists of input data errors and predicted errors. RMSE is the expected error of all samples after taking the input error, which is equivalent to the true error of the interpolation process.

Using absolute error (Mean Bias Error, MBE), Relative Bias Error (RBE), Root-Mean-Square Error (RMSE), RTGCV, Correlation Coefficient (R).As an indicator for evaluating the interpolation effect, the smaller the value, the better the interpolation effect. The specific calculation formula is as follows:


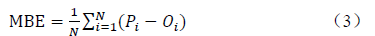


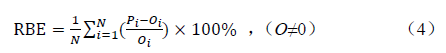


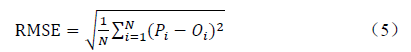


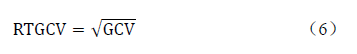


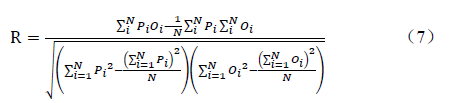


Among them, P_i_ and O_i_ represent the analysis value at the first point and the original observation value of the station, respectively, and N is the number of statistical samples.

1. **Analytical evaluation of dataset**

The ANUSPLIN was used to spatially interpolate the daily and monthly precipitation data of 2472 stations in more than 50 years since 1961, and the precipitation day value and monthly value point data of 0.5°×0.5° were obtained. This paper mainly analyzes and evaluates the high-resolution precipitation grid dataset from the perspective of error analysis, typical case analysis and interpolation test, and provides scientific reference for users.

- 1. **Error Analysis**
     1. Generalized cross-validation

The generalized cross-validation method (GCV) is usually used to verify the effect of interpolation. Figure 3 shows the monthly variation of RTGCV (square root of GCV). It can be seen that in the 600 months since 1961, RTGCV fluctuations have a certain regularity, varying in the range of 0.4-2.2 mm, and have significant periodic characteristics with a period of one year. In order to more clearly present the cyclical changes of RTGCV, Figure 4 shows the monthly change of RTGCV from January to December 2010. According to statistics, the average RTGCV in winter, spring, summer and autumn is 0.6mm and 1.1mm, 1.9mm, 1.2mm, respectively (Table1). Generally, RTGCV reaches the maximum in July and August of summer, and is the smallest in winter and December. By analyzing the spatial distribution of precipitation in the four seasons of winter, spring, summer and autumn (Figure 5), it can be seen that relatively speaking, summer precipitation is generally more, and locality is stronger, and there is no obvious latitude characteristics. Therefore, when the absolute amount of precipitation is large, RTGCV is inevitably relatively large.


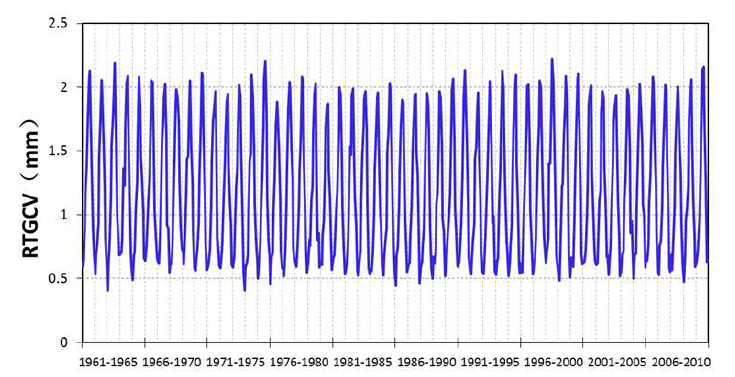


Figure 3 RTGCV changes from January 1961 to December 2010.

Table 1 RTGCV mean and maximum value statistics (mm)

|  | Winter | Spring | Summer | Autumn |
| --- | --- | --- | --- | --- |
| RTGCV_mean_ | 0.6 | 1.1 | 1.9 | 1.2 |
| RTGCV_max_ | 0.8 | 1.5 | 2.2 | 1.9 |
| RTGCV_min_ | 0.4 | 0.6 | 1.4 | 0.5 |


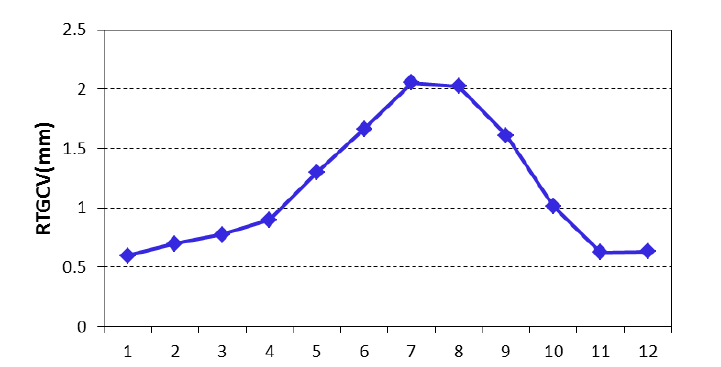


Figure 4 RTGCV changes from January to December 2010.


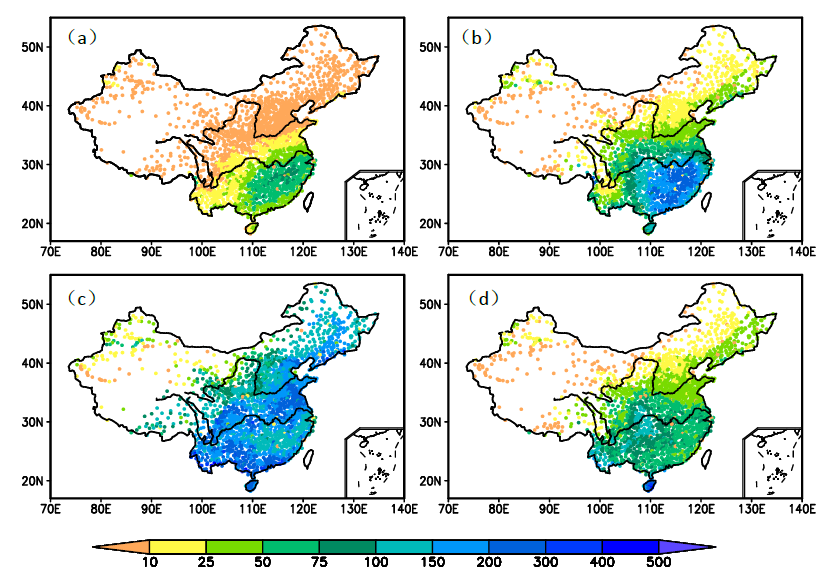


Figure 5 Winter (a), spring (b), summer (c), autumn (d) average precipitation spatial distribution (unit: mm / month).

3.1.2 Root-Mean-Square Error

Root-Mean-Square Error (RMSE) is also one of the commonly used indicators for evaluating interpolation effects. From the monthly curve of RMSE (Figure 6), it can be seen that due to the cyclical variation of precipitation, the RMSE also exhibits a one-year cycle change, which varies from 0.2 mm to 0.8 mm. There have been no obvious interdecadal mutations since 1961. From the analysis of seasonal variation characteristics, RMSE generally reaches the maximum in July and August in summer, and is the smallest in winter and December. This is related to the fact that summer precipitation is high and locality is strong. The average root mean square error of the precipitation grid since 1961 is 0.49mm. It shows that the grid data has better precision and better interpolation effect.


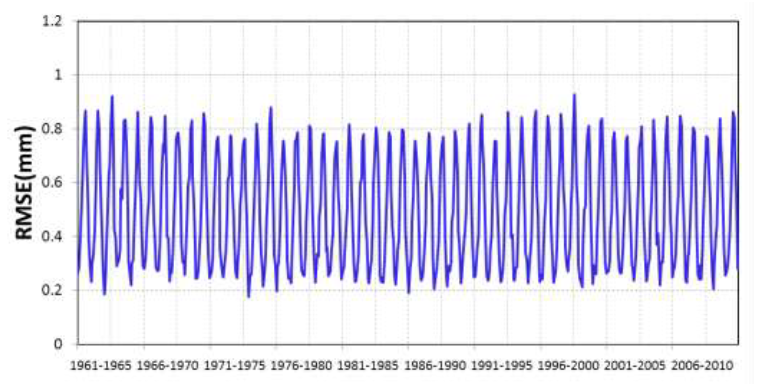


Figure 6 RMSE changes monthly from January 1961 to December 2010.

3.1.3 Absolute error

According to statistics, the correlation coefficient R between the grid analysis value and the site observation value averaged 0.93 (through the significance test of 0.01). Figure 7 shows the spatial distribution of absolute errors between the 600-month national site observations and the corresponding grid analysis values since 1961. It can be seen that no matter which season, the absolute error in the southeast region is mostly higher than other regions. In winter, spring, summer and autumn, the absolute error of 89%, 69%, 30% and 69% stations is within ±3mm/month; the absolute error of 99%, 93%, 67% and 94% stations is ±10mm/ Within the month;

The average errors are -0.1mm/month, -0.3mm/month, -0.8mm/month, -0.3mm/month (Table 2).

Table 2 Absolute error mean and maximum value statistics of each season (mm)

|  | Winter | Spring | Summer | Autumn |
| --- | --- | --- | --- | --- |
| MBE_mean_ | -0.1 | -0.3 | -0.8 | -0.3 |
| MBE_max_ | -0.1 | -0.1 | -0.5 | -0.1 |
| MBE_min_ | -0.2 | -0.6 | -1.1 | -0.7 |


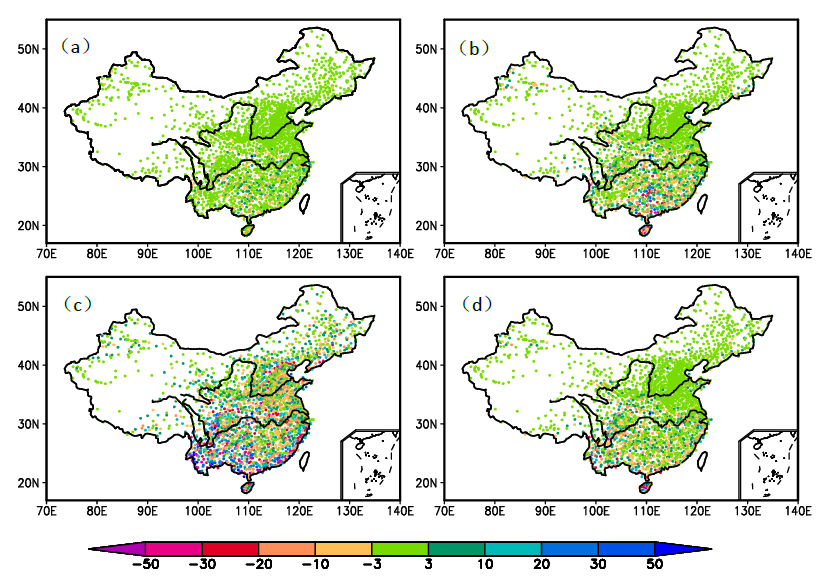


Figure 7 Winter (a), spring (b), summer (c), autumn (d) absolute error spatial distribution (mm / month).

In addition, the frequency distribution of the samples in different absolute error intervals is calculated separately (Figure 8). In 51.5% of the samples, the absolute error between the analytical value and the observed value of the site is within ±5 mm/month; in 75.2% of the samples, the absolute error between the analytical value and the observed value of the site is within ±15 mm/month.


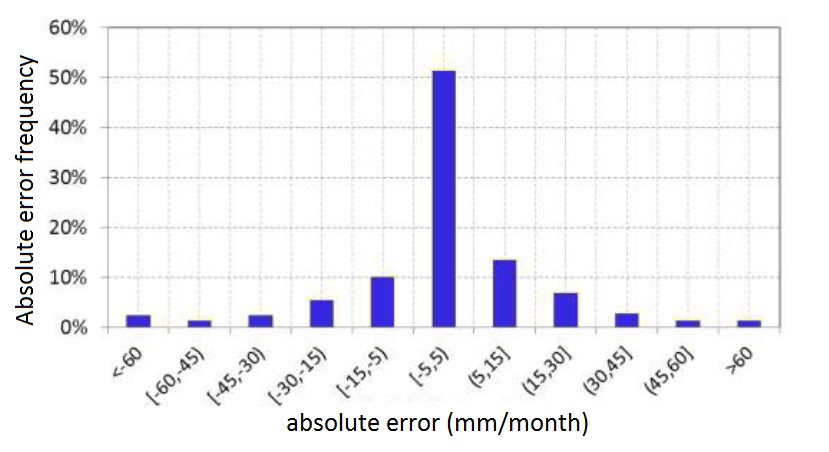


Figure 8 Precipitation monthly value absolute error frequency distribution.

3.1.4 Relative error

For samples with less precipitation, the relative error often cannot objectively reflect the interpolation effect, so this paper only compares the relative error of the sample with monthly precipitation greater than or equal to 10.0mm. Figure 9 shows the spatial distribution of the average relative error of each station since 1961. In winter, the relative error of northwest and northeast China is significantly higher than that of the southeastern region; in summer, the relative error is generally higher than other seasons, but the relative error varies little across the country. In winter, spring, summer and autumn, the relative error of stations of 25%, 51%, 16% and 32% is between ±3%; in winter, spring, summer and autumn, respectively, 60%, 82%, 54%, The relative error of 77% of stations is between ±10% (Figure 10); the national average relative error is -0.2%, 3.3%, 9.1%, 4.5% (see Table 3).


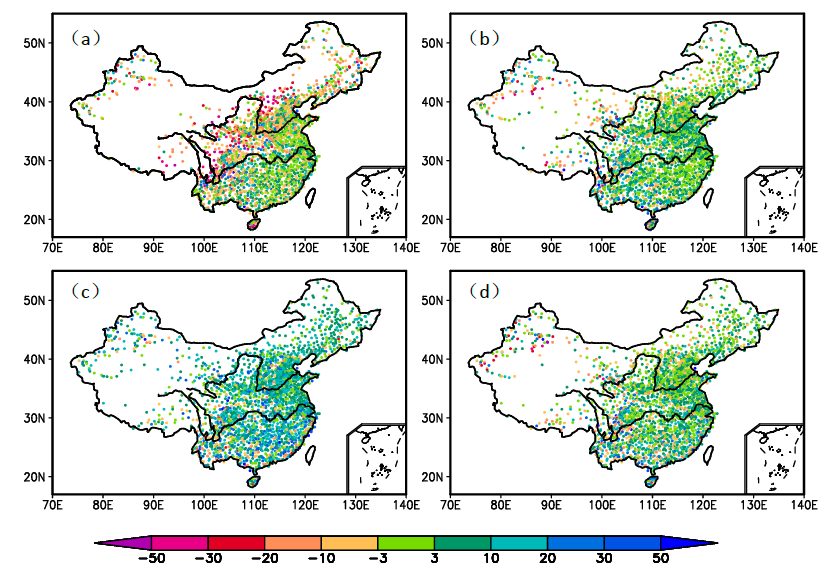


Figure 9 Winter (a), spring (b), summer (c), autumn (d) relative error spatial distribution (unit: %).


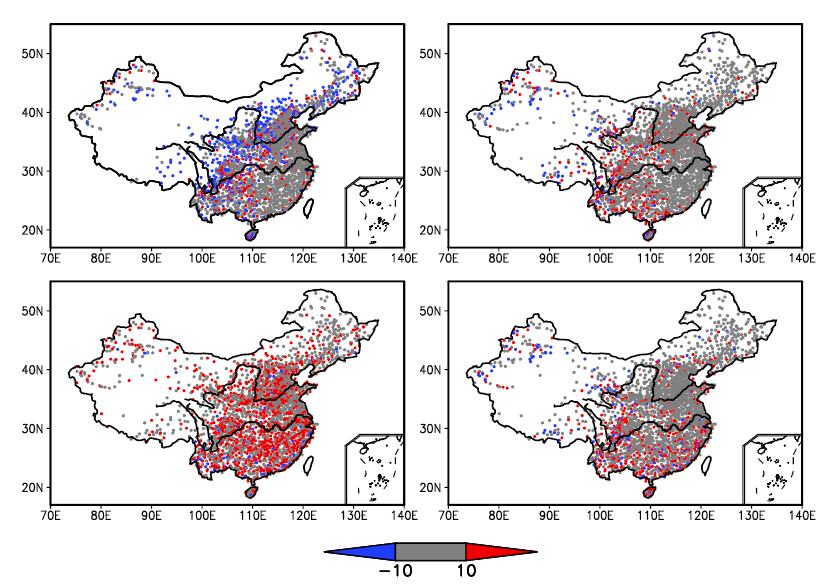


Figure 10 Winter (a), spring (b), summer (c), autumn (d) relative error spatial distribution (unit: %).

Table 3 Relative error mean and maximum value statistics of each season (%)

|  | Winter | Spring | Summer | Autumn |
| --- | --- | --- | --- | --- |
| RMBE_mean_ | -0.2% | 3.3% | 9.1% | 4.5% |
| RMBE_max_ | 1.1% | 6.3% | 11.2% | 8.1% |
| RMBE_min_ | -2.2% | 1.3% | 7.1% | 1.4% |

- 1. **Spatial distribution of climatic conditions**

From the spatial distribution of annual average precipitation in the country from 1961 to 2010 (Figure 11), the interpolated lattice point precipitation data can describe the main spatial characteristics of the annual average precipitation field in the southeast and the northwest in China. It is better to describe the latitudinal variation of annual precipitation, and it also objectively reflects the change of the topographical water with the topography in the north and south of the Tianshan Mountains and the Tarim Basin.


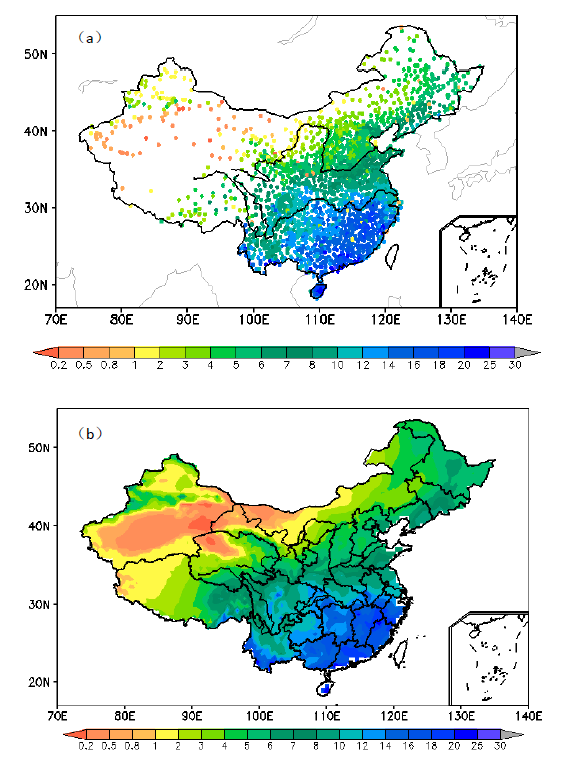


Figure 11 1961-2010 annual average (a) site (b) grid point annual precipitation distribution (100mm).

Summer is a season with more precipitation. It can be seen from Figure 12 that the spatial distribution of summer precipitation through stations and grids can be concluded with the same annual precipitation. The summer precipitation after interpolation can better reflect the main space of China's continental precipitation.


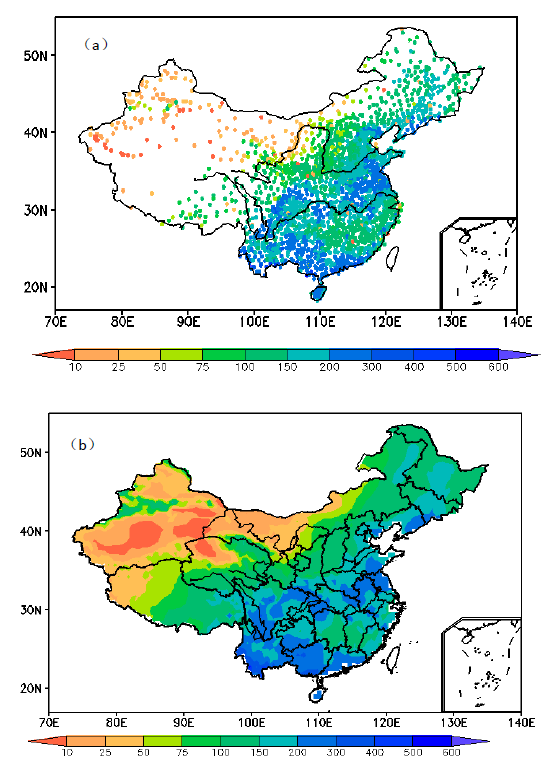


Figure 12 1961-2010 annual average (a) site (b) grid point precipitation distribution in July (mm).

- 1. **Case analysis of surface rainfall**

Surface rainfall refers to the average rainfall over a certain area over a certain period of time. The calculation of surface rainfall in this paper uses the arithmetic mean method. Calculated as follows:


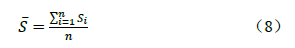


Among them,s is the surface rainfall of the area, the unit is mm (mm); i is the rainfall of the same period in the rainfall measurement station (grid), the unit is mm; represents the number of stations (or grid points).

Figure 13 shows the daily variation of the average RTGCV since 1961. It can be seen that RTGCV is close to 1.2mm at the end of July and early August, reaching the maximum in a year, which is related to the fact that precipitation in China is mainly concentrated in summer.


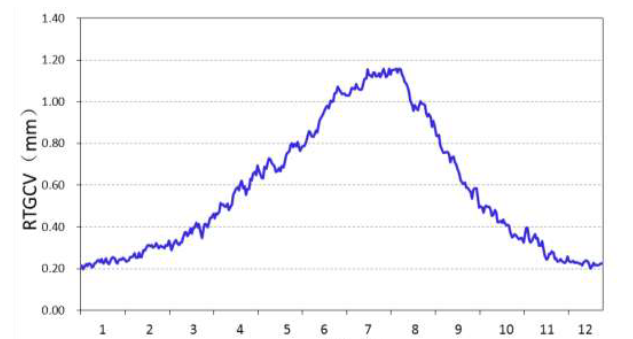


Figure 13 Average RTGCV changes from day to day in 1961-2010.

Therefore, the analysis of surface rainfall is based on the summer precipitation daily value data. This paper selected three examples of heavy precipitation for evaluation and analysis, namely, 2006 Bilis typhoon precipitation, 2009 Morakot typhoon precipitation and July 2012. 21st Beijing heavy precipitation incident. The maximum daily precipitation stations (hereinafter referred to as “strong precipitation stations”) of three heavy precipitation events were selected for interpolation experiments.

According to the precipitation grading standard, a total of 7 test analysis station relative error RBE and surface rainfall relative error RBE were designed. In the seven experiments, the daily precipitation of the heavy precipitation station was assumed to be 250 mm, 100 mm, 50 mm, 25 mm, 10 mm, 0 mm and missing. The average value of the stations and the corresponding grid points are counted in the range of 1, 9, 25, 49, and 81 grid points (0.5° × 0.5°) extending around the strong precipitation station. The amount of surface rain. The test assignment indicates the observation of the daily precipitation of the Boluo station and the relative error of the surface rainfall. The RBE calculation formula is:


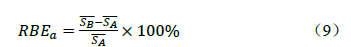


Among them, S_A_ and S_B_ respectively represent the surface rainfall observed by the site and the surface rainfall of the gridded data.

- - 1. Analysis of the Case of Typhoon Precipitation in 2006

Typhoons landing in China will generally bring strong precipitation. In 2006, the typhoon precipitation of Bilis was taken as a case of heavy precipitation, and the effect analysis and interpolation experiment of precipitation daily value point data were carried out. During the typhoon of Bilis, the maximum daily precipitation observation reached 407.6mm, which appeared on the Boluo station in Guangdong on July 15 (114.28°E, 23.18°N). The grid analysis value was 144.2mm. The relative error with the observed value is -64.7%, indicating that the precipitation intensity characterized by the grid analysis value is less than half of the observed value. Since the grid-like interpolation of the station observation data will increase the influence of the observations of nearby neighboring stations, the grid analysis value represents the average precipitation within a certain range. Figure 14 shows the spatial distribution of observations and grid values ​​for precipitation sites on July 15, 2006. The relative rainfall error RBE of the lattice unit number of 1 (M=1) is -45.9%, while the RBE of M=9 is only 5.6%, and when M=25, the RBE is 28.0%. Compared with M=9, there is a large increase. When M=49 and M=81, the RBE is 16.7% and 11.9%, respectively. It can be seen that due to the strong local characteristics of precipitation, in this heavy precipitation event, when M=9, the relative error of surface rainfall is minimized.


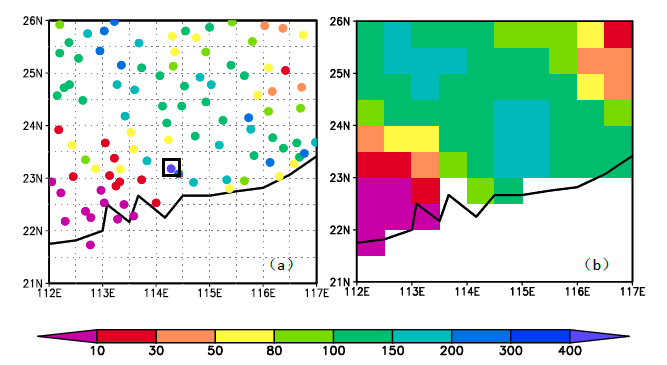


Figure 14 Precipitation observations (a) and grid points (b) spatial distribution on July 15, 2006 (the square station in Guangdong is Boluo Station, Guangdong; unit: mm).

- - 1. Analysis of the Cases of Heavy Rainfall in Beijing at 7.21 in 2012

On July 21, 2012, Beijing suffered from strong precipitation in the past 61 years and received widespread attention from all walks of life. Since the data source for the development of “China Ground Precipitation 0.5°×0.5° Grid Dataset (V2.0)” includes only 2472 national-level stations, the largest daily precipitation observation on July 21 is 290.9mm. At Xiayunling Station (115.73°E, 39.73°N), the grid analysis value is 118.9mm, and the relative error between the analytical value and the observed value of the station is -59.2%, which indicates that the precipitation intensity characterized by the grid analysis value is significantly higher than that of the observation. Weak. The grid point value can more accurately describe the spatial characteristics of heavy precipitation centered on the Beijing area (Figure 16). The surface rainfall relative to the error RBE is 1.3% average when the number of cells is 1 (M = 1), and the MBE is -14.6 and -16.6% when M=9 and M=25, compared with M= 1 has a large increase, and RBE is -0.3% and 1.1% when M=49 and M=81, respectively. It can be seen that in this heavy precipitation event, when M=49, the relative error of surface rainfall is minimized.


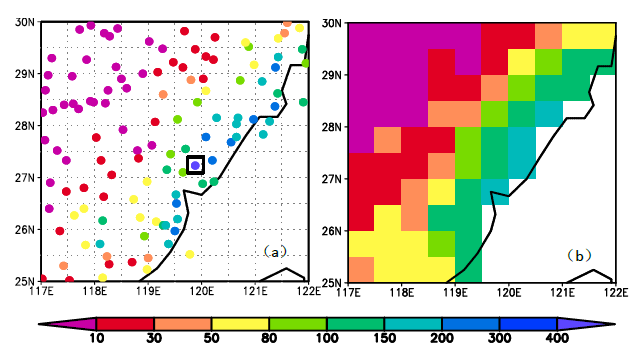


Figure 15 Precipitation observations (a) and grid points (b) spatial distribution on August 9, 2009 (the square station in Fujian is Fujian Yongrong Station; unit: mm).


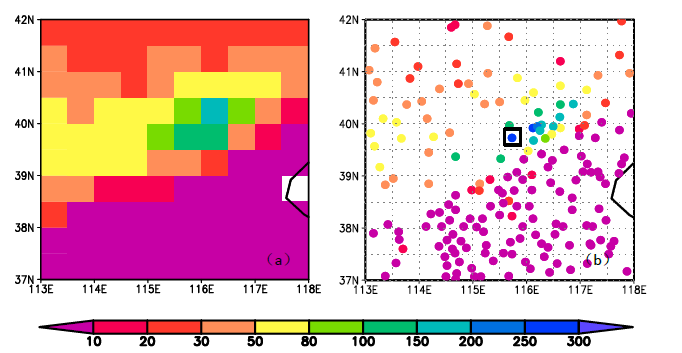


Figure 16 July 21, 2012 precipitation observations (a) and grid points (b) spatial distribution (square grid station is Beijing Xiayunling station; unit: mm).

Through the analysis of the above two typhoon strong precipitation cases and the 7.21 strong precipitation events in Beijing, the results show that:

Due to the strong precipitation brought by typhoon registration, it is often concentrated in individual stations. Therefore, the precipitation with the largest precipitation has a large difference with the surrounding stations. The relative rainfall error is larger when M=1, and the surface rainfall is M=9. The relative error is the smallest; in the event of heavy rainfall in Beijing, the locality of precipitation is weaker than that of typhoon, and the station density is large. The relative error of surface rainfall is small when M=1, and the relative error of surface rainfall is M=49. Achieve the minimum.

The above interpolation experiments are based on individual heavy precipitation events, which inevitably have certain limitations, but the following conclusions can still be drawn: Although grid processing has certain difficulties in accurately depicting precipitation anomalies (such as large precipitation), However, when considering a certain range of surface rain, the difference between the grid analysis value and the observation value is small, and the grid point precipitation can well describe the magnitude of the surface rainfall within a certain range.

**4. Summary**

Through a comprehensive data quality of "China ground precipitation 0.5 ° × 0.5 ° grid data set (V2.0)"

The quantity assessment mainly leads to the following conclusions:

(1) RTGCV fluctuations have a certain regularity, varying in the range of 0.4-2.2mm, and have significant periodic characteristics. Because summer precipitation is generally more and more local, there is no obvious latitude characteristics. Therefore, when the absolute amount of precipitation is large, RTGCV will inevitably be too large.

(2) The average root mean square error of the precipitation grid point is 0.49 mm. It shows that the grid data has better precision and better interpolation effect.

(3) In winter, spring, summer and autumn, the absolute error of 89%, 69%, 30% and 69% stations is within ±3mm/month; the absolute error of 99%, 93%, 67% and 94% stations is Within ±10mm/month; the average error is -0.1mm/month, -0.3mm/month, -0.8mm/month, -0.3mm/month.

(4) In winter, spring, summer and autumn, the relative error of stations of 25%, 51%, 16% and 32% is between ±3%; 60%, 82%, 54%, 77% of stations The relative error is between ±10%; the national average relative error is -0.2%, 3.3%, 9.1%, and 4.5%, respectively.

(5) It can be seen from the spatial distribution of the annual average precipitation climatic state that the interpolated lattice point precipitation data can describe the main spatial characteristics of the annual average precipitation field in the southeast and the northwest in China in a detailed and accurate manner.

In summary, the lattice analysis value is highly correlated with the original sequence, and the RTGCV and root mean square errors are small. Since 1961, China's ground precipitation daily value and monthly value grid data can accurately reflect the variation characteristics of precipitation. From the perspective of surface rainfall, the analysis of precipitation cases shows that although grid processing has certain difficulty in accurately depicting precipitation anomalies (such as large precipitation), when considering the amount of surface rainfall within a certain range, the grid analysis value The difference from the observed value is small, and the lattice precipitation can well describe the magnitude of the surface rainfall within a certain range.

**Reference:**

[1] Hutchinson, M. F., Interpolation of Rainfall Data with Thin Plate Smoothing Splines - Part I:Two Dimensional Smoothing of Data with Short Range Correlation. Journal of Geographic Information and Decision Analysis, vol. 2, no. 2, pp. 139-151, 1998.

[2] Hutchinson, M. F., Interpolation of Rainfall Data with Thin Plate Smoothing Splines - Part II: Analysis of Topographic Dependence Journal of Geographic Information and Decision Analysis, vol. 2, no. 2, pp. 152-167, 1998.

[3] Hutchinson, M. F., Gessler P E. 1994. Splines more than just a smooth interpolator. Geoderma, 62 (1):45-67.

[4] Hutchinson M F. 1995. Interpolating mean rainfall using thin plate smoothing splines. Int J GIS ,9 (4) :385-403.

[5] Hutchinson M F. 1991. The application of thin plate smoothing splines to continent2wide data assimilation. Jasper J D. BMRC Research Report No. 27 , Data Assimilation Systems. Melbourne : Bureau of Meteorology , 1042113.


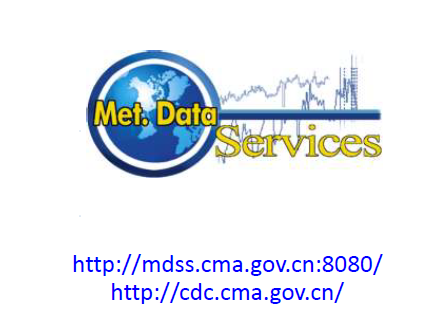


**Contact Unit: National Meteorological Information Center Data Service Room**

**Address: 46 South Street, Zhongguancun, Haidian District, Beijing**

**Postal Code: 100081**

**On duty: 010-68407499**

**010-68406106**

**Fax: 010-62175930**

**Mailbox: cdc@cma.gov.cn**
