# Supplementary material for: Analysis and prediction of vegetation dynamics under the background of climate change in Xinjiang, China
Source: PeerJ. 2020 Jan 23;8:e8282. doi: 10.7717/peerj.8282 (PMC6983299; doi:10.7717/peerj.8282)
Supplement: Supplemental Information 1 [file peerj-08-8282-s001.zip › Introduction of data/Intruduction of Meteorological dataset metadata.docx]

**Meteorological dataset metadata**

**Dataset identification information**

**Dataset name:** China ground precipitation daily value 0.5 ° × 0.5 ° grid data set (V2.0)

**Dataset code:** SURF_CLI_CHN_PRE_DAY_GRID_0.5

**Abstract:** This dataset is based on the latest data of the China Ground High-density Station (2472 national meteorological observatories) compiled by the National Meteorological Information Center. The thin disk spline method (TPS, Thin Plate Spline) using ANUSPLIN software. Spatial interpolation is performed to generate daily precipitation map data from 1961 to the latest Chinese ground level resolution of 0.5° × 0.5°.

**Data quality**

Data quality description:

The data is cross-validated, error analysis, and the quality is in good condition.

Data processing process:

Step 1: Using the data from January 1, 1961 to the latest precipitation date value, format conversion to generate data in accordance with the requirements of the ANUSPLIN software;

Step 2: Using GTOPO30 data, after framing decompression, format conversion, resampling, etc., generate digital elevation model data dem of 0.5°×0.5° in China;

Step 3: Run the selnot.exe module in the ANUSPLIN software to select the initial node according to the generalized cross-validation value optimization principle;

Step 4: Select three independent variables of longitude, latitude, and altitude, run the splinb.exe module, and generate a surface coefficient file.

Step 5: Run the lapgrd.exe module to generate daily 0.5°×0.5° precipitation grid data;

Step 6: Perform cross-validation and error analysis on the precipitation grid dataset.

**Data source:**

The data source of the data set includes two parts: the daily precipitation data of the national stations (basic, benchmark and general stations) from 1961 to the latest national collections collected by the National Meteorological Information Center; A digital elevation model DEM of China's land 0.5° × 0.5° produced by re-sampling from GTOPO30 data (resolution 0.05° × 0.05°).

**Dataset classification:**

Ground meteorological data

**Update frequency:**

Regular

**Key words:**

Subject classification keywords: surface meteorological data, precipitation

Geographical Range Keywords: China Land

Hierarchical Keywords: Ground, Precipitation, Grid

**Spatial resolution:**

0.5° × 0.5°

**Reference system:**

None

**Time stamp:**

Production time:

2012.08.01

Production type:

Production

**Geographic coverage**

Geographical Description: China

The most west longitude: 72 °E

The most east longitude: 136 °E

Northernmost latitude: 54°N

Southernmost latitude: 18°N

**Time coverage:**

Starting time: 19610101

Termination time: latest

Observation or statistical frequency: day by day

**Sharing level:** 1

**Contact method:**

Dataset owner name: Zhao Yufei

Dataset responsible unit name: National Meteorological Information Center data service room

DataSet Responsible Officer: Senior Engineer

DataSet Responsible Person Role Code: Producer

Contact information

Tel: (010)68407499

 Fax: (010)68407499

Detailed address: National Meteorological Information Center Data Service Room

City: Beijing

Administrative District: Beijing

Postal code: 100081

Country: China

E-mail: [cdc@cma.gov.cn](mailto:cdc@cma.gov.cn)

**Metadata entity information**

Metadata ID: SURF_CLI_CHN_PRE_DAY_GRID_0.5

Metadata Language: Chinese

Metadata character set: universal character set

Metadata production date: 20120801

Metadata standard adopted: core metadata of meteorological data set (QX/T 39-2005)

Metadata Standard Version: 1.0
