# Supplementary material for: Analysis and prediction of vegetation dynamics under the background of climate change in Xinjiang, China
Source: PeerJ. 2020 Jan 23;8:e8282. doi: 10.7717/peerj.8282 (PMC6983299; doi:10.7717/peerj.8282)
Supplement: Supplemental Information 1 [file peerj-08-8282-s001.zip › Introduction of data/Meteorological dataset documentation.docx]

**Meteorological dataset documentation**

1. **Dataset information**

Dataset Chinese name: China ground precipitation daily value 0.5 ° × 0.5 ° grid data set (V2.0)

Dataset code: SURF_CLI_CHN_PRE_DAY_GRID_0.5

Dataset version: V2.0

Dataset establishment time: 20120801

1. **Data source**

The data source of the dataset includes two parts: the daily precipitation value of the national stations (basic, benchmark and general stations) from 1961 to the latest national collections collected by the National Meteorological Information Center. Data; digital elevation model DEM of China land 0.5°×0.5° produced by re-sampling from GTOPO30 data (resolution 0.05°×0.05°).

1. **Dataset entity**

3.1. Dataset Entity Description

3.1.1. Dataset Entity File Name

China precipitation daily value 0.5 ° × 0.5 ° grid data set file naming consists of data set code (SURF_CLI_CHN_PRE_DAY_GRID_0.5), year and month date identification (YYYYMMDD).

Specific form: SURF_CLI_CHN_PRE_DAY_GRID_0.5-YYYYMMDD.TXT

- - 1. Description of the contents of the data set entity file

The data set storage format is ARCGIS standard format, and the data set entity includes January 1st, 1961 - to the latest daily data file, and the first 6 behavior header files included in each file, wherein:

The first line "ncols 128" indicates that the entity data has 128 columns;

The second line "nrows 72" indicates that the entity data has 72 lines;

The third line "xllcorner 72" indicates that the longitude range of the bottom left cell of the data is 72°-72.5°E;

The fourth line "yllcorner 18" indicates that the latitude range of the bottom left grid unit of the data is 18°-18.5°N;

The fifth line "cellsize 0.5" means that the grid is 0.5° × 0.5°;

The sixth line "NODATA_value -9999.0" indicates that the value outside the Chinese region is represented by -9999.0.

Starting from the seventh line is the precipitation value of the corresponding grid, and the seventh row (the first row of precipitation data) is the center of the first column of the data grid (72.25°E, 53.75°N), and the seventh row and the second column of the data grid. The center is (72.75°E, 53.75°N), ..., and the last column of the last row of the data is centered at (135.75°E, 18.25°N). The precipitation value is reserved for 1 decimal place. Longitude unit: degree, latitude unit: degree, grid point precipitation unit: mm.

- - 1. Description of eigenvalues

Values outside the Chinese region are indicated by -9999.0.

3.2. Data storage information

3.2.1. Storage format and reading

The data set storage format is ARCGIS standard format, text file, fixed long record, and read by line.

3.2.2. Placement of data sets in media

Storage directory structure:

Datasets: Stores dataset entity files. A total of 18840 daily grid point precipitation data files from January 1, 1961 to July 31, 2012 were included.

Metadata document (SURF_CLI_CHN_PRE_DAY_GRID_0.5_META_C.doc).

Description: Description document (SURF_CLI_CHN_PRE_ DAY_GRID_0.5_DOCU_C.DOC).

Documents: holds the dataset attached files.

3.3. Time attribute

Time range: January 1, 1961 to the latest

Time resolution: day by day

3.4. Spatial properties

3.4.1. Geographical scope

Geographical Description: China

The most west longitude: 72 °E

The most east longitude: 136 °E

Northernmost latitude: 54°N

Southernmost latitude: 18°N

3.4.2. Station Information: None

3.4.3. Spatial resolution: 0.5° × 0.5°

3.4.4. Vertical range: none

3.4.5. Projection: None

3.5. Observation instruments: See the relevant content of the Ground Observation Specification for details.

3.6. Data processing method

Step 1: Using the data from January 1, 1961 to the latest precipitation date value, format conversion to generate data in accordance with the requirements of the ANUSPLIN software;

Step 2: Using GTOPO30 data, after framing decompression, format conversion, resampling, etc., generate digital elevation model data dem of 0.5°×0.5° in China;

Step 3: Run the selnot.exe module in the ANUSPLIN software to select the initial node according to the generalized cross-validation value optimization principle;

Step 4: Select three independent variables of longitude, latitude, and altitude, run the splinb.exe module, and generate a surface coefficient file.

Step 5: Run the lapgrd.exe module to generate daily 0.5°×0.5° precipitation grid data;

Step 6: Perform cross-validation and error analysis on the precipitation grid dataset.

3.7. Data quality status

3.7.1 Quality Control Method: Cross Validation Quality Test Method

3.7.2 Quality status: The data set is cross-validated and error analysis, and the quality is in good condition.

3.8. Data Integrity: Includes January 1, 1961 - the latest data file, complete with data.

**4. References:**

[1] Hutchinson, M. F., Interpolation of Rainfall Data with Thin Plate Smoothing Splines - Part I: Two Dimensional Smoothing of Data with Short Range Correlation. Journal of Geographic Information and Decision Analysis, vol. 2, no. 2, pp. 139-151, 1998.

[2] Hutchinson, M. F., Interpolation of Rainfall Data with Thin Plate Smoothing Splines - Part II: Analysis of Topographic Dependence Journal of Geographic Information and Decision Analysis, vol. 2, no. 2, pp. 152-167, 1998.

**5. Data set production and technical support**

5.1. Dataset producer

Name: Zhao Weifei Zhu Jiang Xu Yan Liu Na

Unit: National Meteorological Information Center Information Service Room

5.2. Dataset Document Compiler

Name: Zhao Yufei

Unit: National Meteorological Information Center Information Service Room

5.3. Technical support

Unit: National Meteorological Information Center Information Service Room

Phone: 68074499

Fax: 68074499

E-mail: cdc@cma.gov.cn

Postal code: 100081

Address: No. 46, South Street, Zhongguancun, Haidian District, Beijing

**6. Other: None**
