# Supplementary material for: Analysis and prediction of vegetation dynamics under the background of climate change in Xinjiang, China
Source: PeerJ. 2020 Jan 23;8:e8282. doi: 10.7717/peerj.8282 (PMC6983299; doi:10.7717/peerj.8282)
Supplement: Supplemental Information 2 [file peerj-08-8282-s002.zip › Declaration.docx]

**Declaration**

All the interpolation results take up a lot of storage space and the file cannot be uploaded. So we choose a few representative file uploads.

The information of pictures are as follows:

Temperature: tem

Unit: 0.1℃

Precipitation: pre

Unit: 0.1mm
