# Supplementary material for: Analysis and prediction of vegetation dynamics under the background of climate change in Xinjiang, China
Source: PeerJ. 2020 Jan 23;8:e8282. doi: 10.7717/peerj.8282 (PMC6983299; doi:10.7717/peerj.8282)
Supplement: Supplemental Information 3 [file peerj-08-8282-s003.docx]

- ***L119 The accuracy of the extended dataset, and should be added in suppinfo file.***

“L119 The soil moisture dataset of GLDAS 2.0 has been extended to 2018 by a linear regression model.”

**Response:**

The soil moisture data at different depths, net longwave radiation, net shortwave radiation, and surface specific humidity were collected from the Global Land Data Assimilation System (GLDAS). The GLDAS dataset was valeted against available data from multiple sources. In this study, we used GLDAS 2.0 data (NOAH, 0.25°× 0.25°) for the period 1980-2010 and GLDAS 2.1 data (NOAH, 0.25°× 0.25°) from 2001 to 2018. We built a linear relationship model to extend the GLDAS-2.0 dataset to 2018 by using data from two datasets overlap periods.

- ***L151-154 should be provided in the supp file, how you produce based on temporal resolution.***

“L151-154 We applied the Maximum Value Composites (MVC) method to synthesize monthly and annual data. To ensure the consistency of these data, this study established a pixel-by-pixel linear regression model to interpolate different sensor data during the repetition period”

**Response:**
